# Supplementary material for: Synthesis and Spectroscopic Characterization of Selected Phenothiazines and Phenazines Rationalized Based on DFT Calculation
Source: Molecules. 2022 Nov 4;27(21):7519. doi: 10.3390/molecules27217519 (PMC9653876; doi:10.3390/molecules27217519)
Supplement: Supplementary file 1 [file molecules-27-07519-s001.zip › molecules-1978911-supplementary.pdf]

Supporting Information for

Synthesis and Spectroscopic Characterization of Selected Phenothiazines and Phenazines Rationalized Based on DFT calculation

Daniel Swoboda[1], Jacek E. Nycz[1,*] Nataliya Karaush-Karmazin[2], Boris Minaev[2], Maria Książek[3], Joachim Kusz[3] and Radosław Podsiadły[4]

[1] Institute of Chemistry, Faculty of Science and Technology, University of Silesia in Katowice, ul. Szkolna 9; PL-40007 Katowice, Poland
[2] Department of Chemistry and Nanomaterials Science, Bohdan Khmelnytsky National University, 18031 Cherkasy, Ukraine
[3] Institute of Physics, Faculty of Science and Technology, University of Silesia in Katowice, 75 Pułku Piechoty 1a, 41-500 Chorzów, Poland
[4] Institute of Polymer and Dye Technology, Faculty of Chemistry, Lodz University of Technology, Stefanowskiego 12/16, 90-924, Lodz, Poland
* Correspondence: jacek.nycz@us.edu.pl; Tel.: +48-32-359-1446

Table of Contents

Figures S1–S6. The optimized molecular structures of molecules **1, 2a**, **3, 5** and **6a** in the S_0 and S_1 states

Figures S7–S8. TDDFT/B3LYP/6-31G(d,p) simulated absorption spectra of molecules **2a** and **3**

Figures S9–S10. Molecular orbitals for the molecules **2a** and **3** calculated at the TDDFT/B3LYP/6-31G(d,p) level of theory.

Figure S11. The structure and energies of the two possible conformers for **2a**.

Figures S12a-f. ^{1}H, ^{13}C{^{1}H} NMR; MS and HRMS spectra of the **2a**.

Figures S13a-d. ^{1}H, ^{13}C{^{1}H} NMR; MS and HRMS spectra of the **5**.

Figures S14a-d. ^{1}H, ^{13}C{^{1}H} NMR; MS and HRMS spectra of the **6a**.

Figures S15a-d. ^{1}H, ^{13}C{^{1}H} NMR; MS and HRMS spectra of the **6b**.

Figures S16a-d. ^{1}H, ^{13}C{^{1}H} NMR; MS and HRMS spectra of the **6c**.

Figures S17a-d. ^{1}H, ^{13}C{^{1}H} NMR; MS and HRMS spectra of the **6d**.

Figures S18a-d. ^{1}H, ^{13}C{^{1}H} NMR; MS and HRMS spectra of the **6e**.

Table S1. Wavelengths (λ), oscillator strengths (*f*) and orbital assignment of the selected electronic transitions in the absorption spectra of molecules **2a** and **3** calculated at the TDDFT/B3LYP/6-31G(d,p) level of theory.

Table S2. Spectroscopic data of the S_1–S_0 electronic transition in the emission spectra of compounds **2a** and **3**.

Table S3. Crystal data and structure refinement details of compounds **1, 2a**, **2b**, **3, 5** and **6a**.

Tables S4–S9. Geometric parameters for **1**, **2a**, **2b**, **3**, **5**, **6a**.

Table S10. The experimental aromatic ^{1}H chemical shifts of compounds **1**, **2**, **3**, **5** and **6**.

Table S11. The experimental aromatic ^{13}C{^{1}H} chemical shifts of compounds **1**, **2**, **3**, **5** and **6**.

Tables S12-S16. Optimized Cartesian coordinates of the **1**, **2a**, **3**, **5** and **6a.**

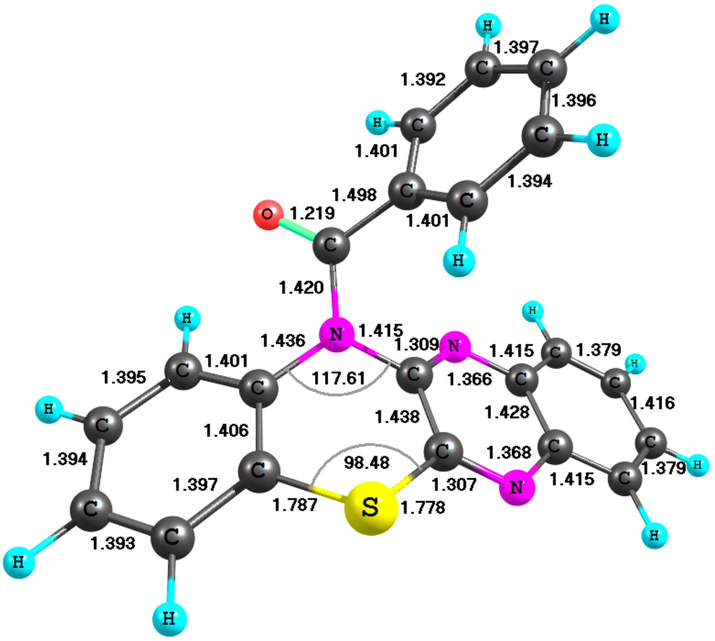

a(S_0 state)

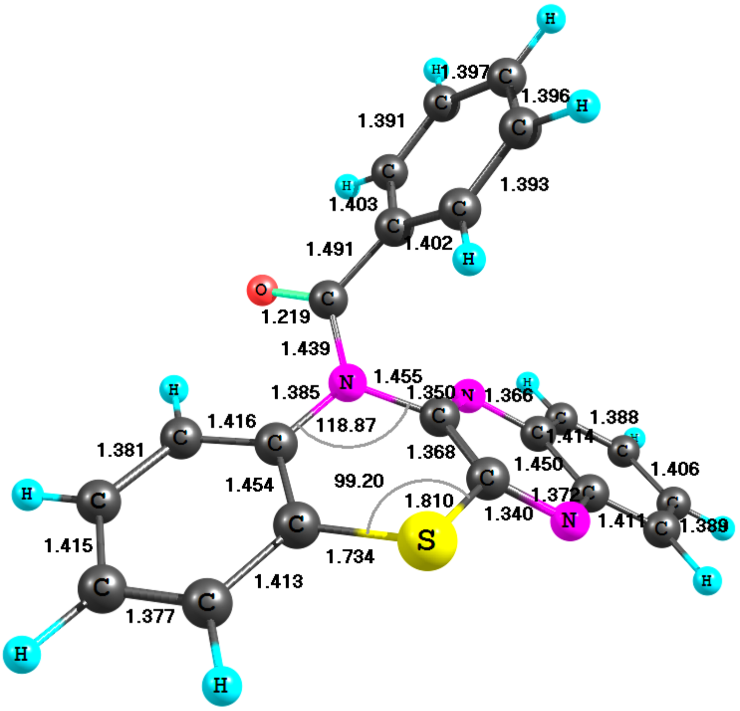

b(S_1 state)

Figure S1. The optimized molecular structure and selected bond lengths (Å) and angles (°) of the 1 in the S_0 (*a*) and S_1 (*b*) states calculated at the B3LYP/6-31G(d,p) level of theory

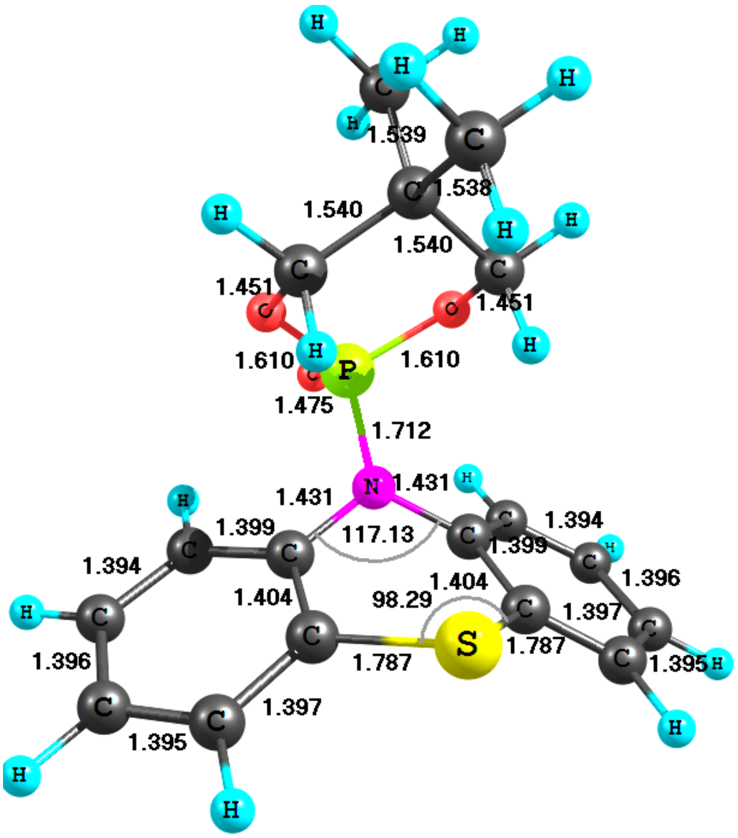

a(S_0 state)

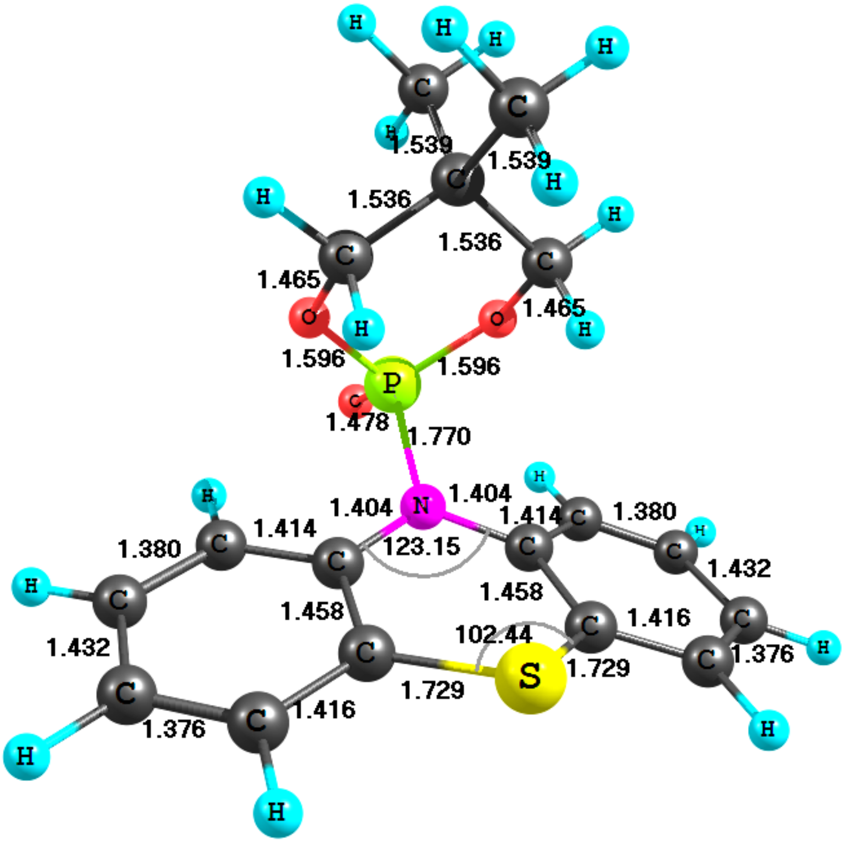

b(S_1 state)

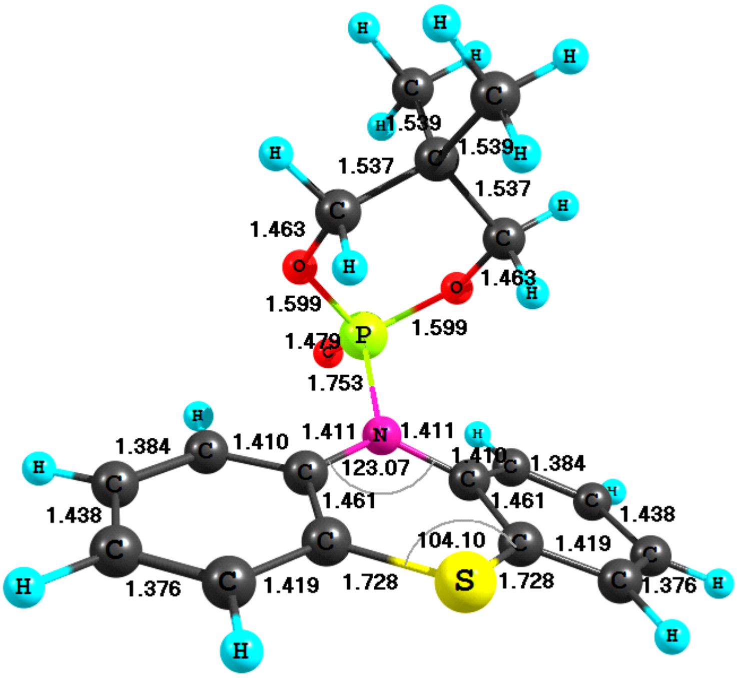

c (T_1 state)

Figure S2. The optimized molecular structure and selected bond lengths (Å) and angles (°) of the 2a in the S_0 (*a*), S_1 (*b*) and T_1 (*c*) states calculated at the B3LYP/6-31G(d,p) level of theory

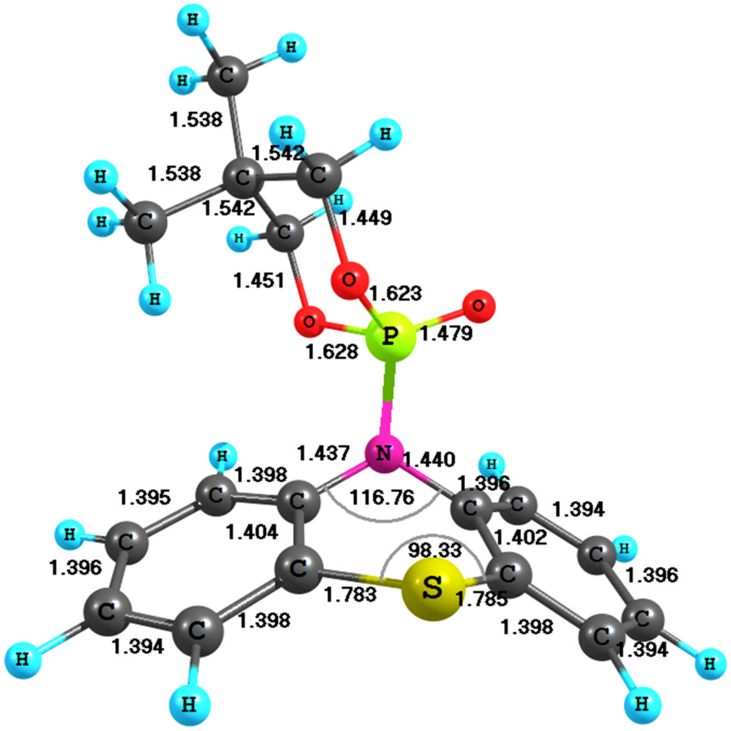

Figure S3. The optimized molecular structure and selected bond lengths (Å) and angles (°) of the 2a conformer in the S_0 state calculated at the B3LYP/6-31G(d,p) level of theory

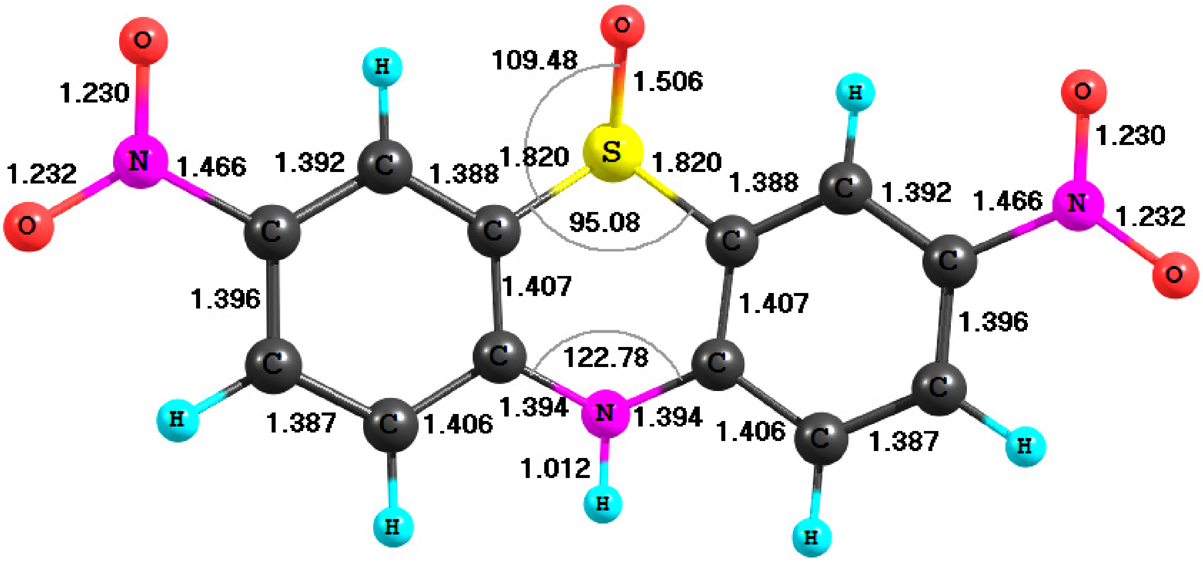

a(S₀ state)

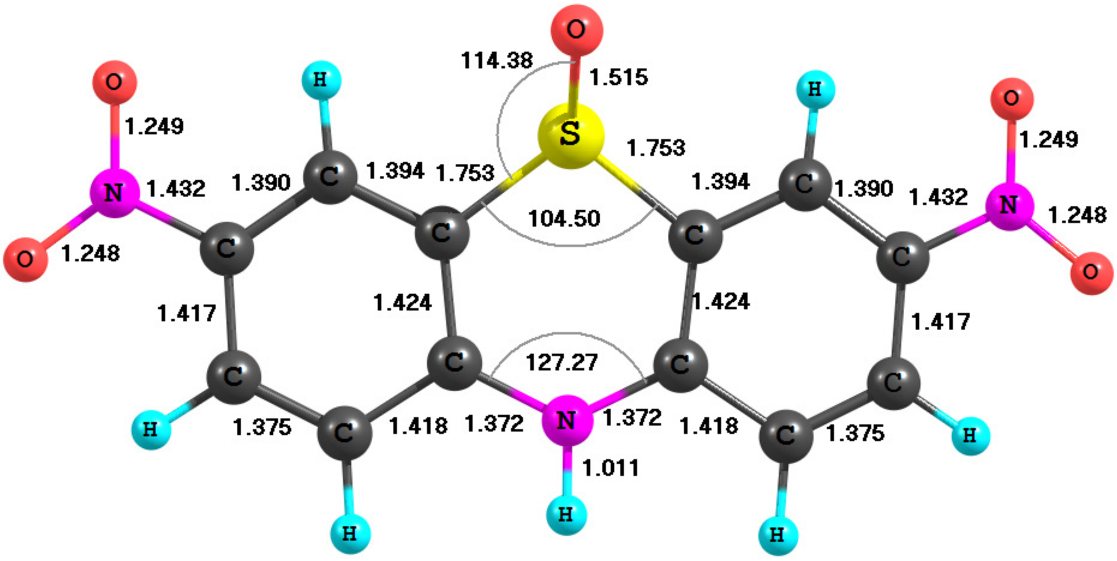

b(S₁ state)

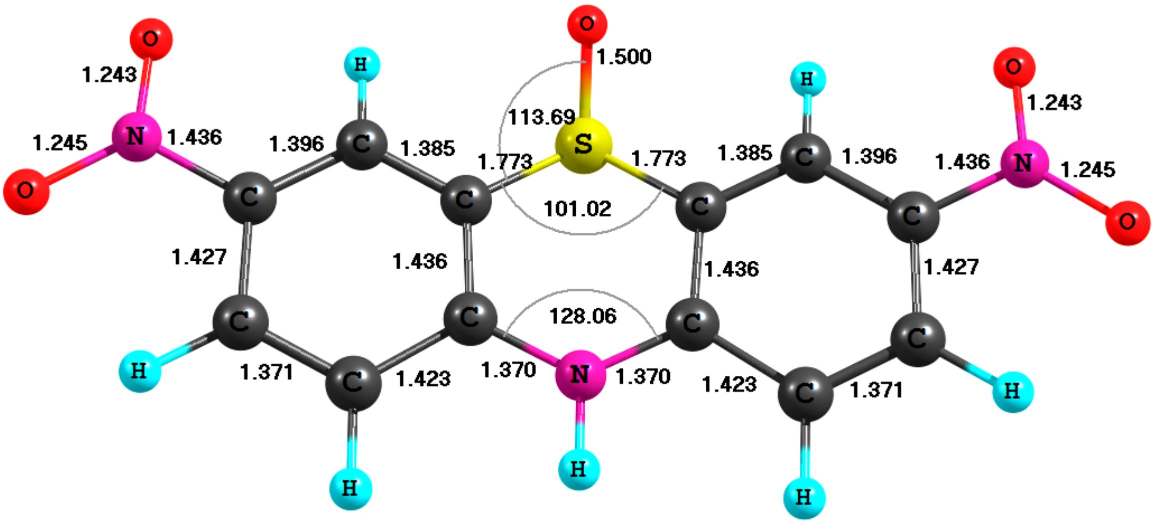

c (T₁ state)

Figure S4. The optimized molecular structure and selected bond lengths (Å) and angles (°) of the 3 in the S_0 (*a*), S_1 (*b*) and T_1 (*c*) states calculated at the B3LYP/6-31G(d,p) level of theory

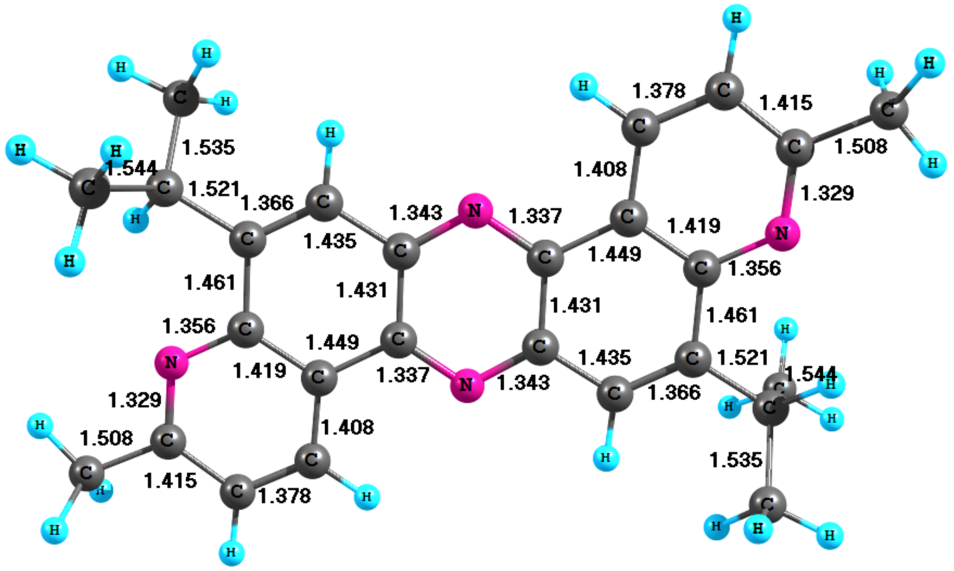

a(S₀ state)

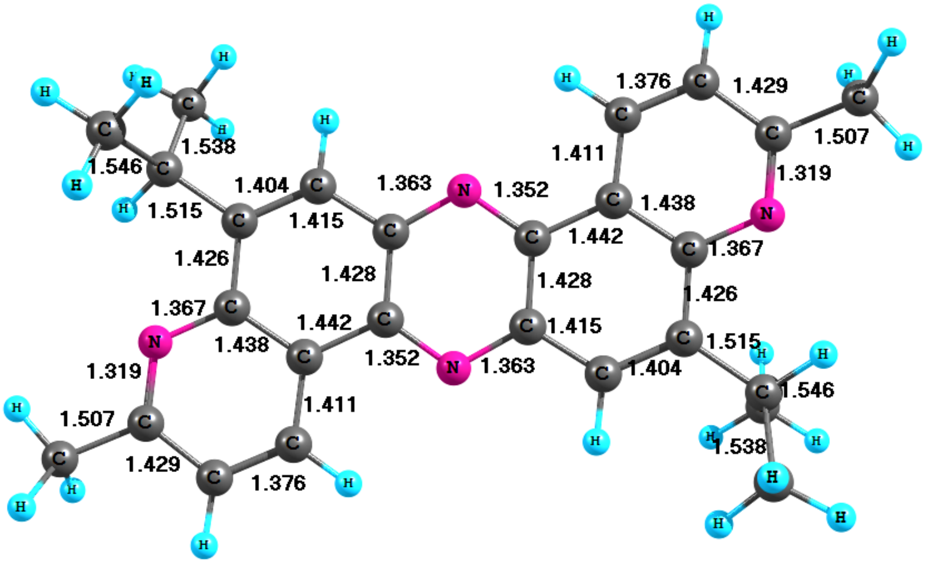

b(S₁ state)

Figure S5. The optimized molecular structure and selected bond lengths (Å) of the 5 in the S_0 (*a*) and S_1 (*b*) states calculated at the B3LYP/6-31G(d,p) level of theory

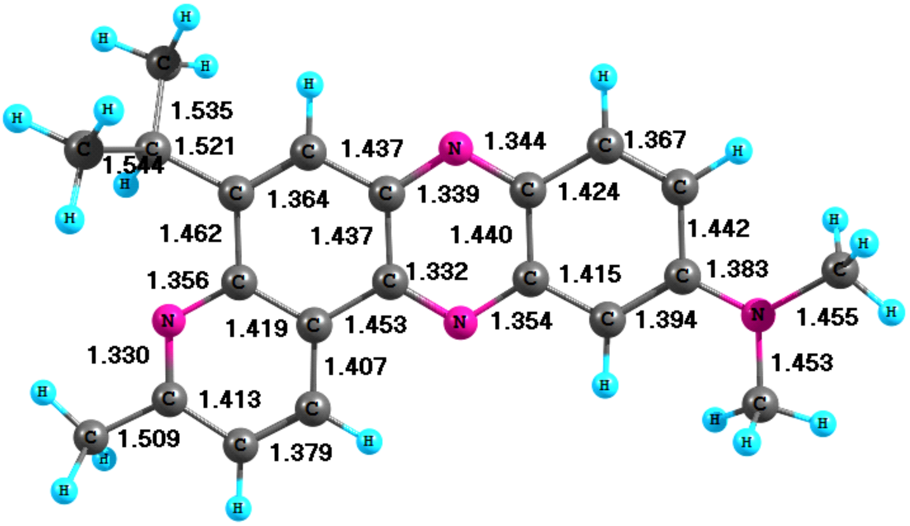

a(S_0 state)

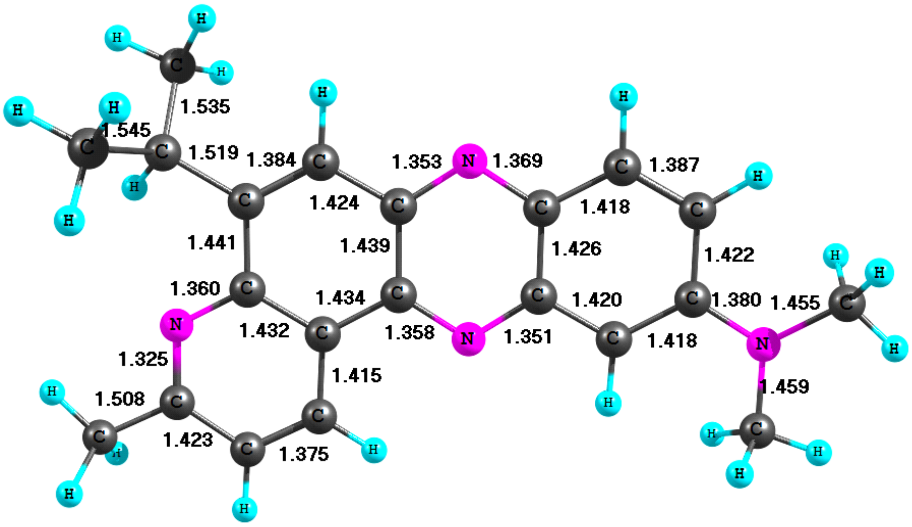

b (S_1 state)

Figure S6. The optimized molecular structure and selected bond lengths (Å) of the 6a in the S_0 (*a*) and S_1 (*b*) states calculated at the B3LYP/6-31G(d,p) level of theory

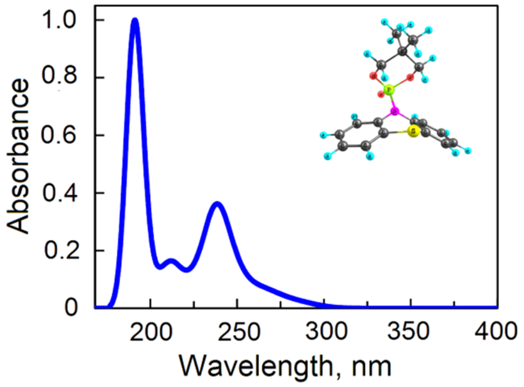

Figure S7. B3LYP/6-31G(d,p) simulated absorption spectra of the compound **2a** taking into account solvent effects (solvent – MeCN).

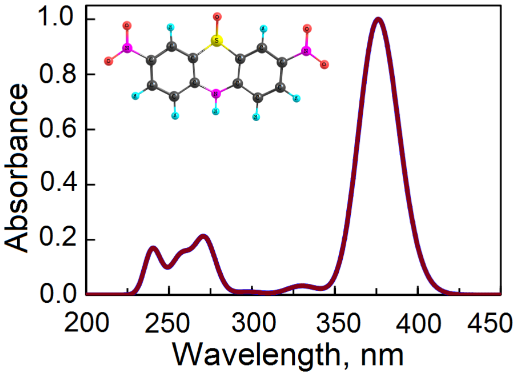

Figure S8. B3LYP/6-31G(d,p) simulated absorption spectra of the 3,7-dinitro-10*H*-phenothiazine 5-oxide (**3**) taking into account solvent effects (solvent – dimethylsulfoxide).

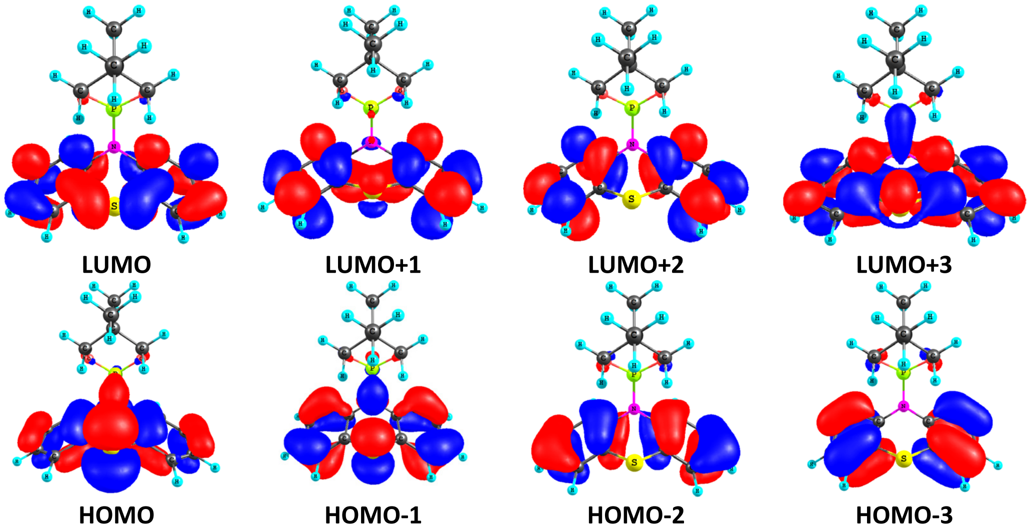

Figure S9. Molecular orbitals for **2a** calculated at the B3LYP/6-31G(d,p) level of theory.

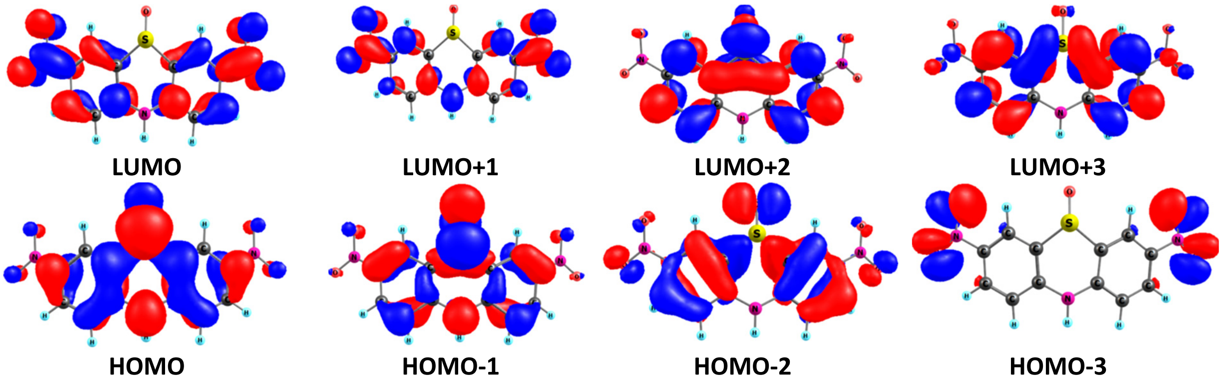

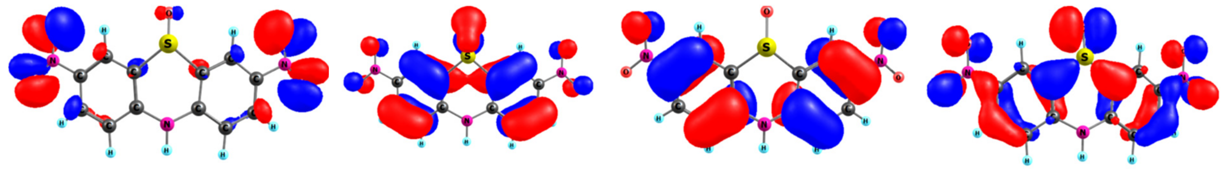

HOMO-4 **HOMO-5** **HOMO-6** **HOMO-7**

Figure S10. Molecular orbitals for **3** calculated at the B3LYP/6-31G(d,p) level of theory.

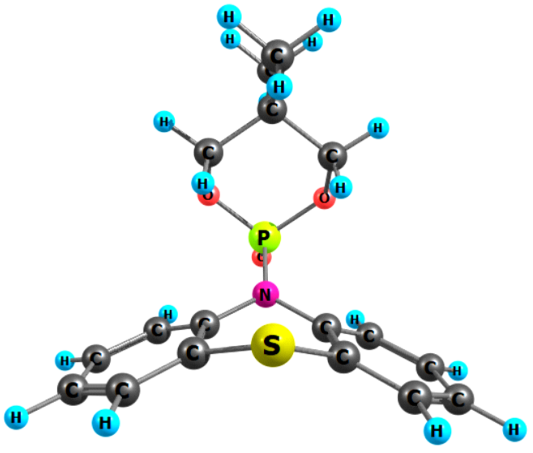	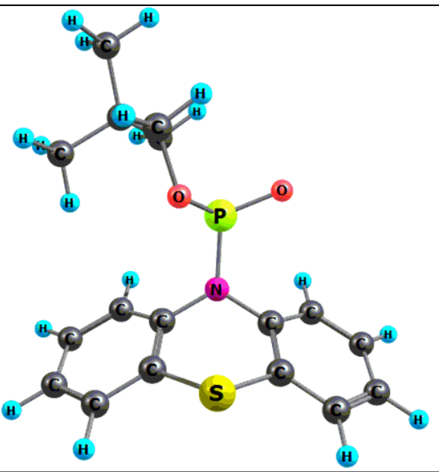
**front view**	**front view**
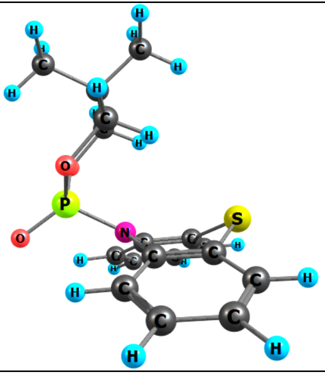	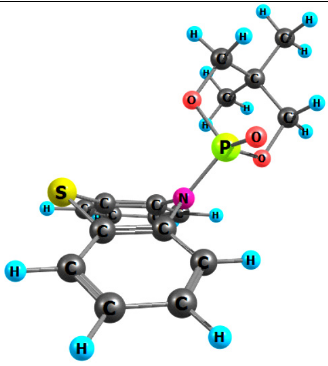
**side view**	**side view**
**2a (chair conformer 1)**<br>E(B3LYP/6-31G(d,p)) =-1678.71849516 a.u.	**2a (chair conformer 2)**<br>E(B3LYP/6-31G(d,p)) = -1678.71618296 a.u.

Figure S11. The structure and energies of the two possible conformers (gas-phase B3LYP/6-31G(d,p) calculation).

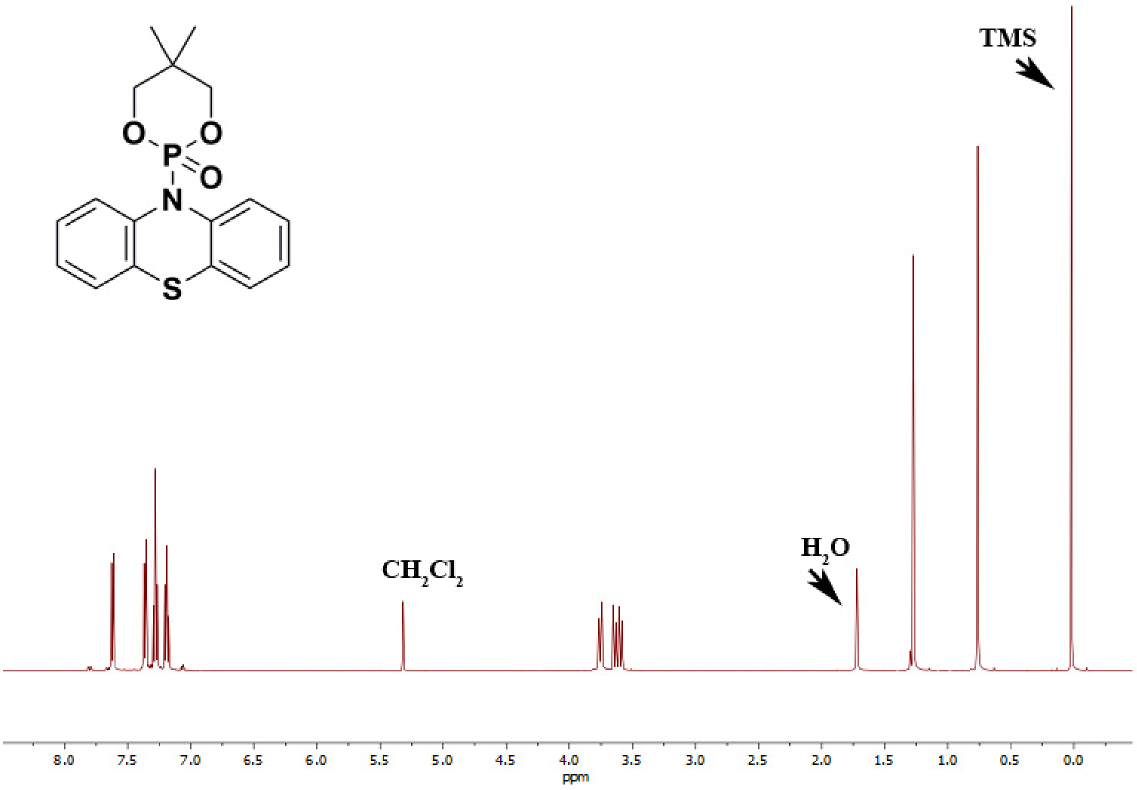

Figure S12a.^{1}H-NMR ($CDCl_3$; 500.2 MHz) spectrum of the **2a**.

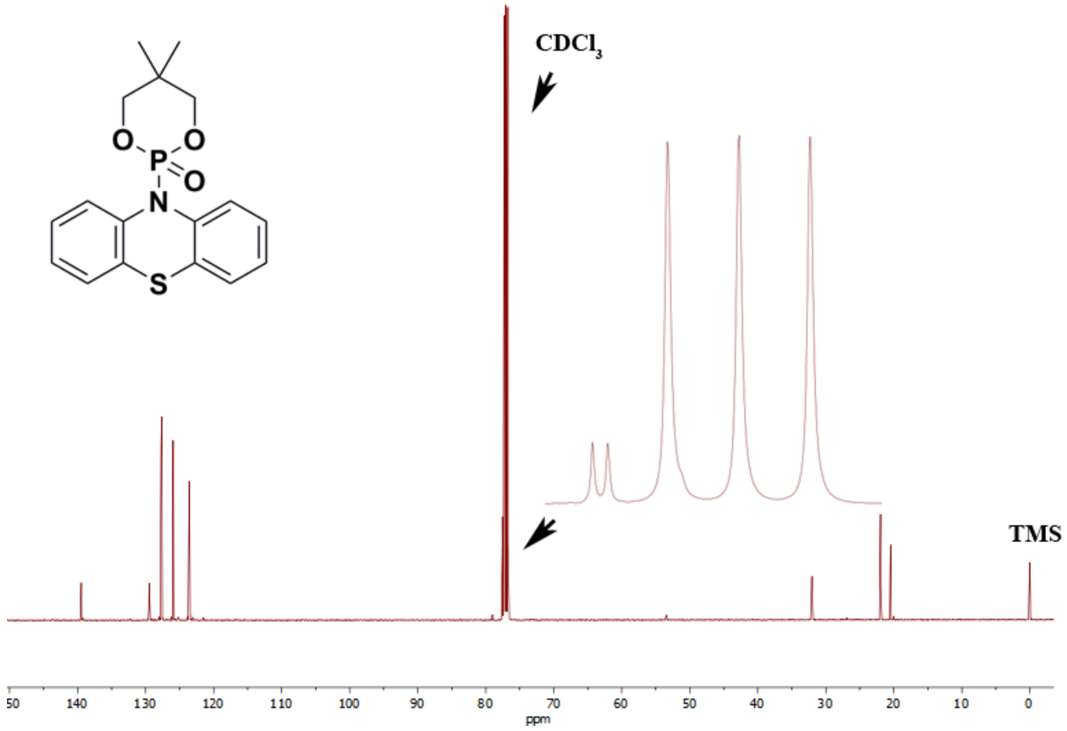

Figure S12b.$^{13}C\{^1H\}$-NMR ($CDCl_3$; 125.8 MHz) spectrum of the **2a**.

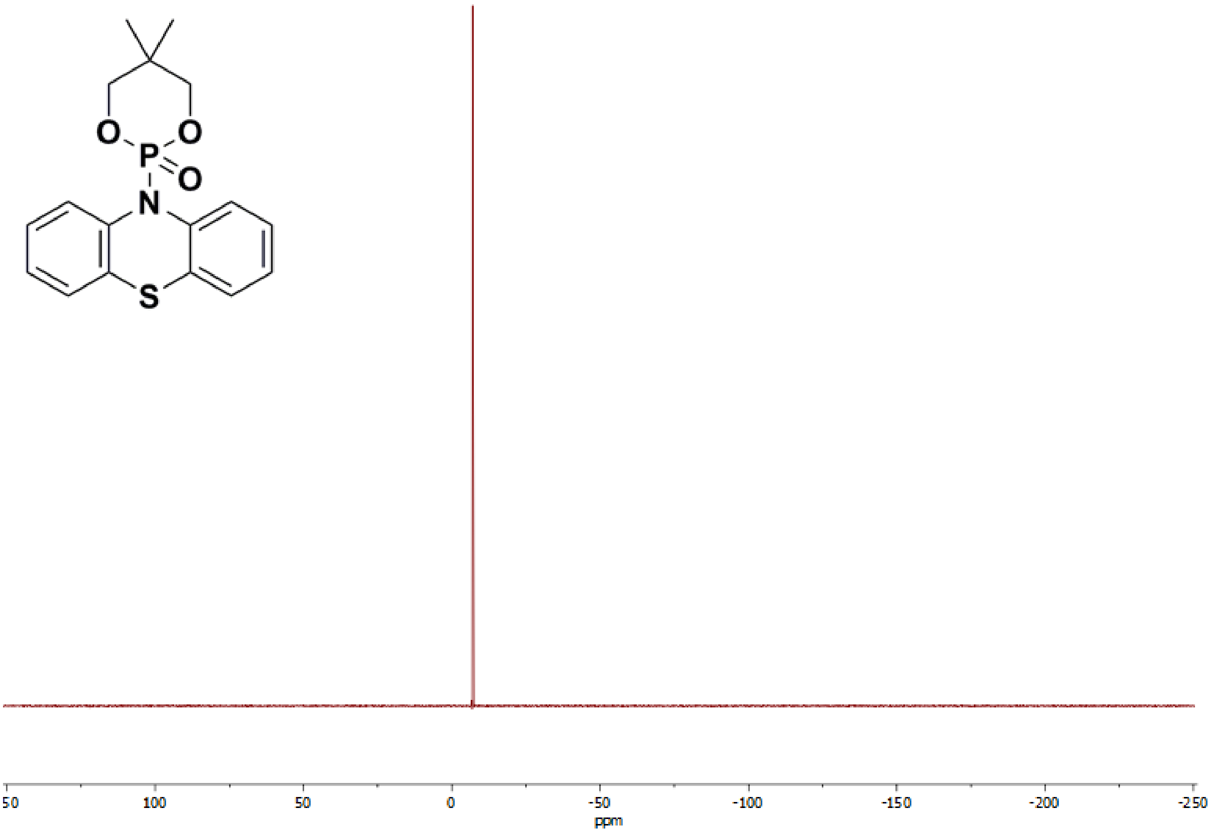

Figure S12c. $^{31}P\{^{1}H\}$-NMR ($CDCl_3$; 202.5 MHz) spectrum of the **2a**.

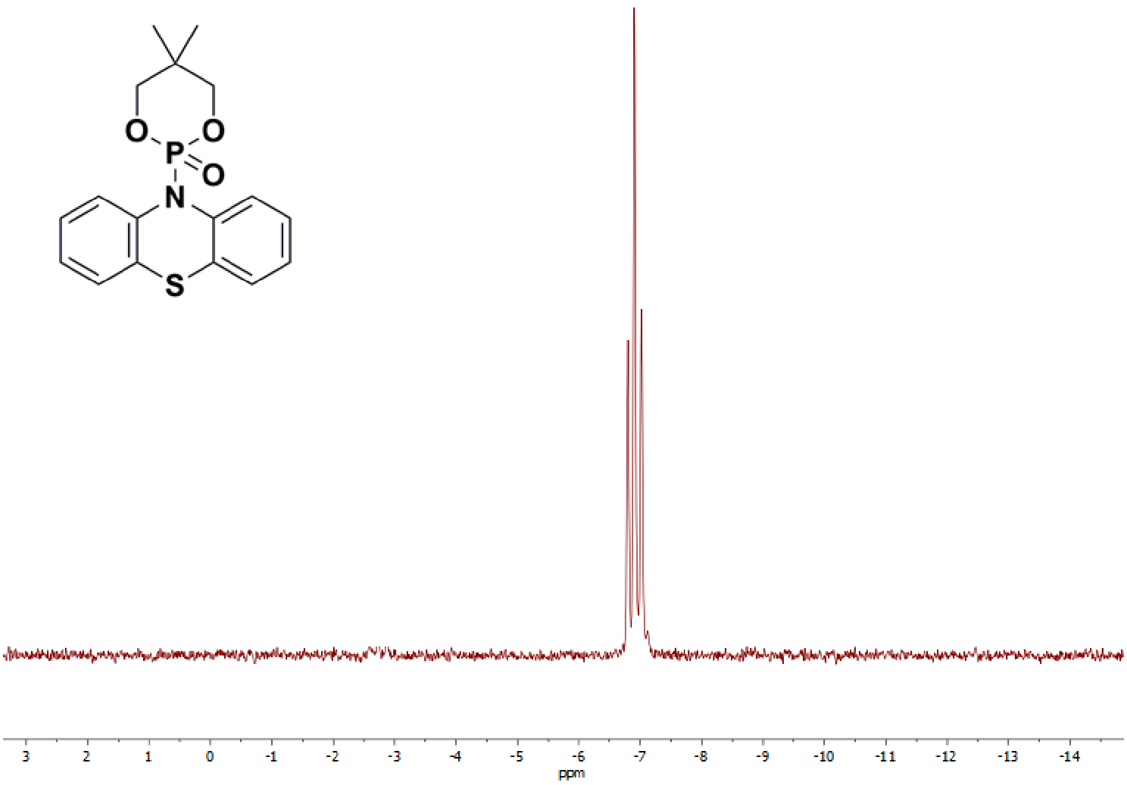

Figure S12d. ^{31}P-NMR ($CDCl_3$; 202.5 MHz) spectrum of the **2a**.

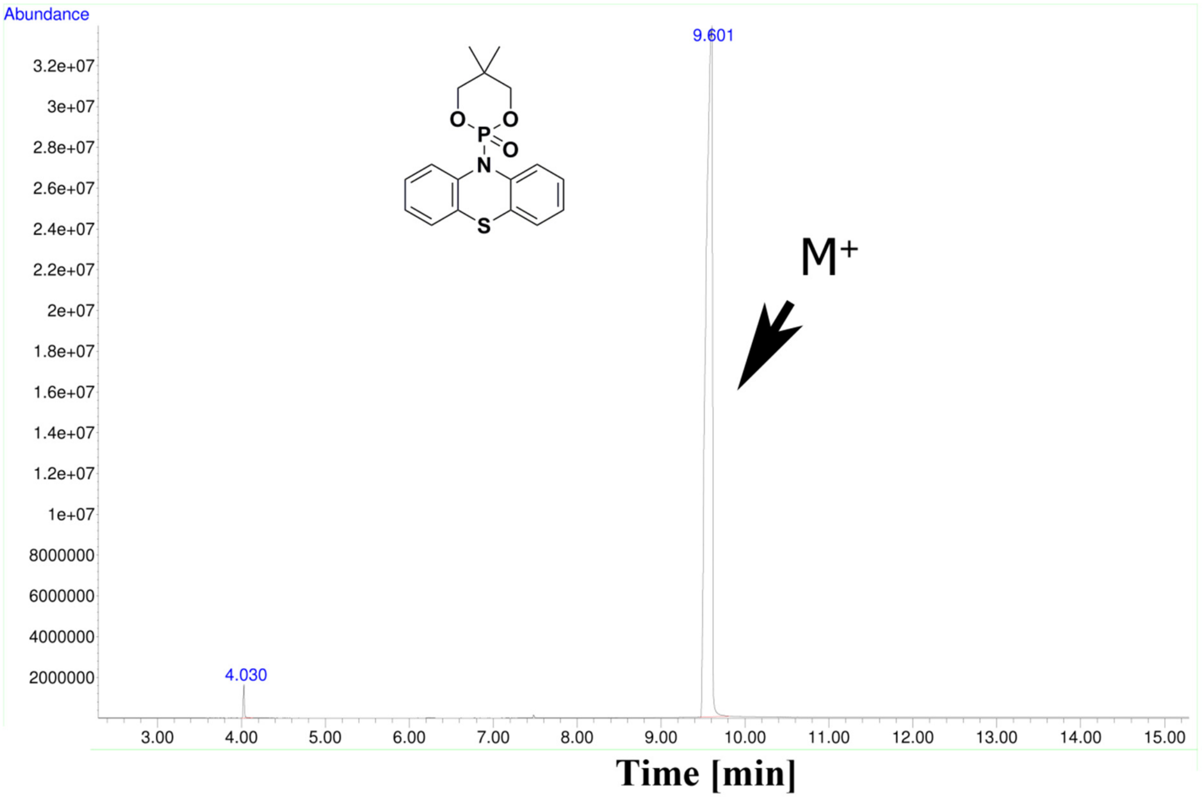

Figure S12e. Chromatogram of the molecule **2a**.

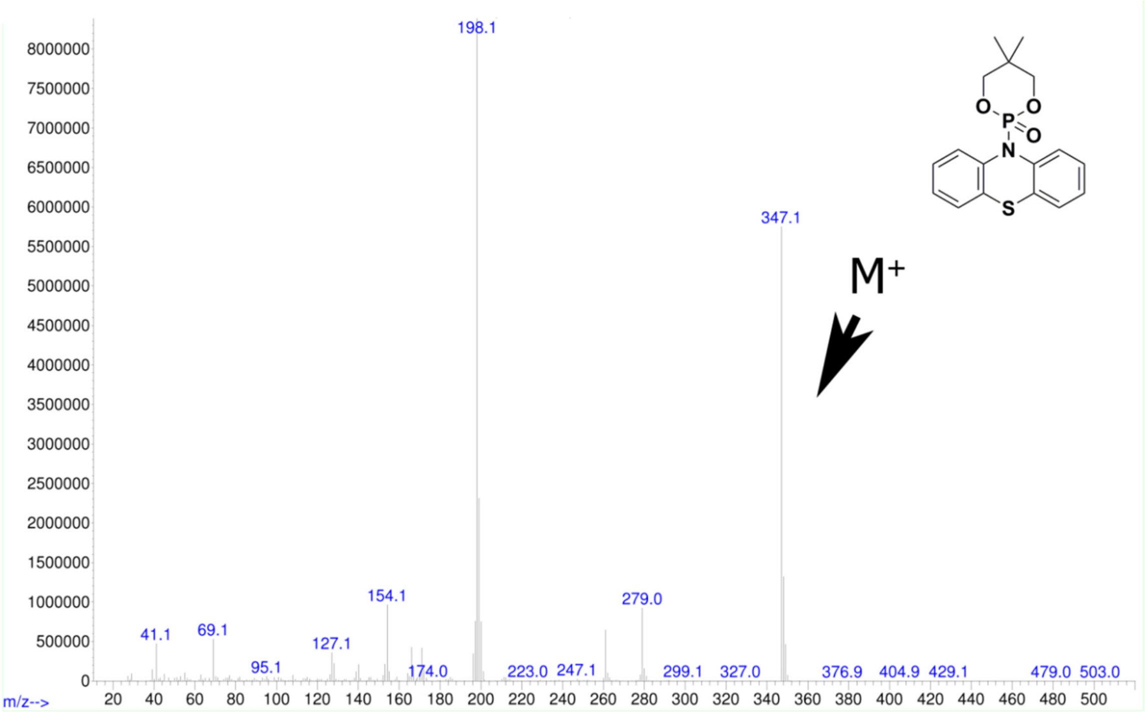

Figure S12f. MS spectrum of the molecule **2a**.

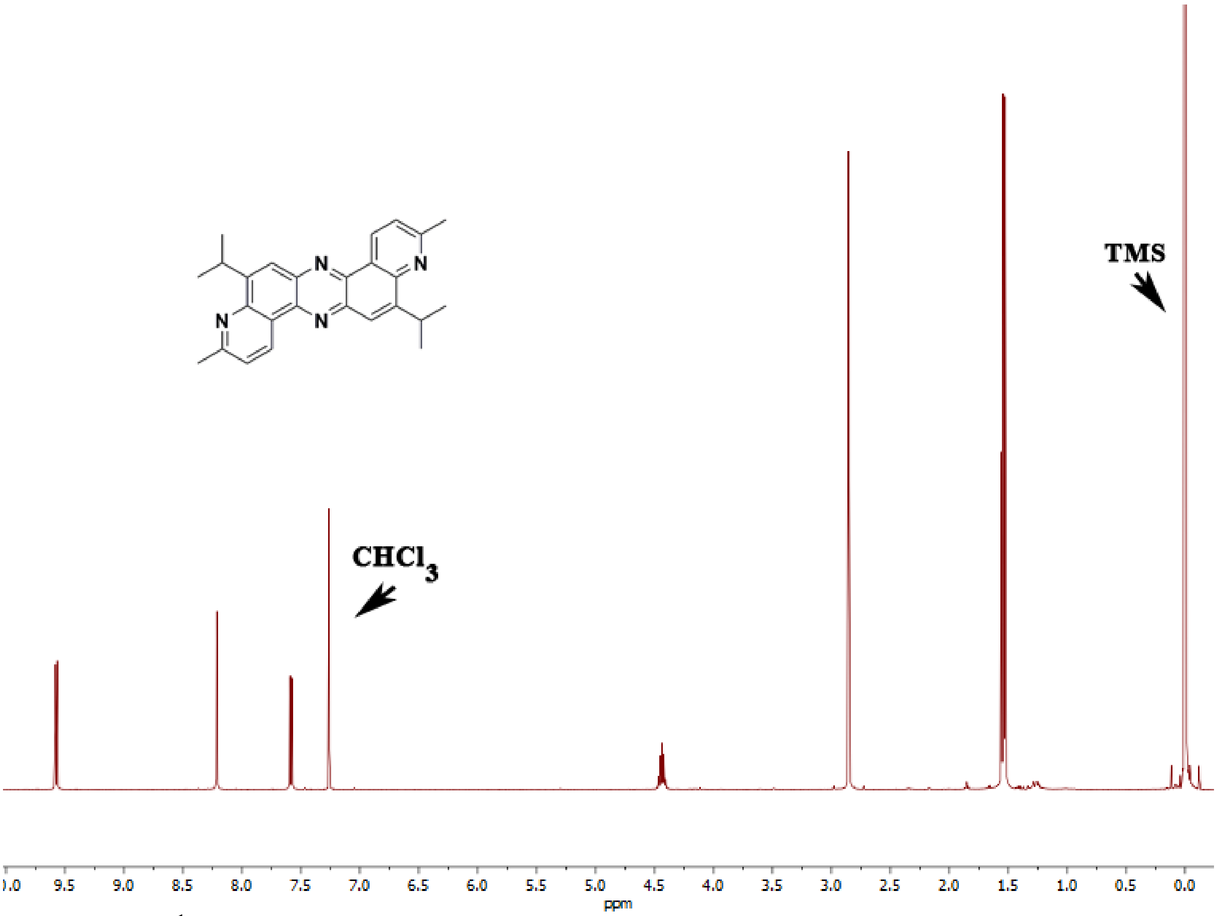

Figure S13a. ^{1}H-NMR ($CDCl_3$; 500.2 MHz) spectrum of the **5**.

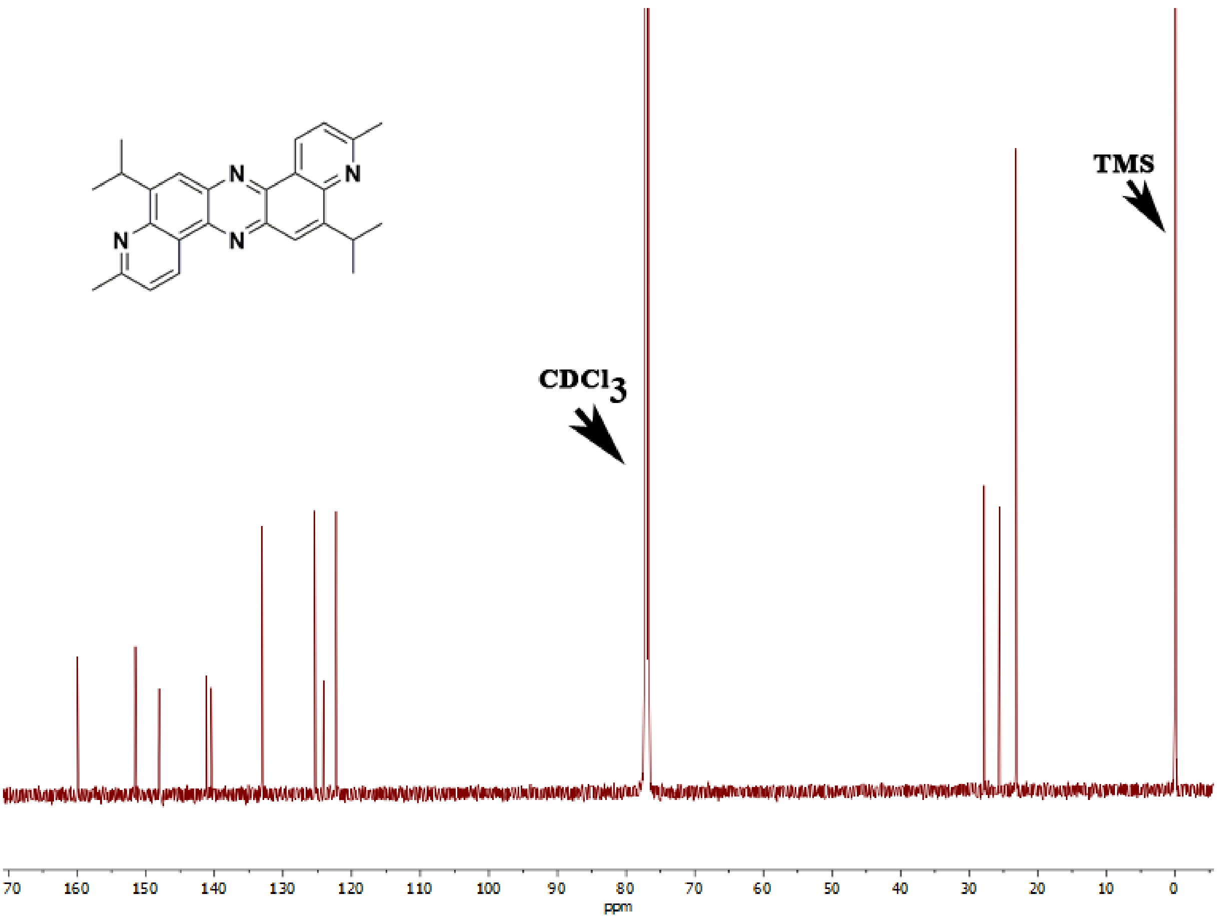

Figure S13b.$^{13}C\{^{1}H\}$-NMR ($CDCl_3$; 125.8 MHz) spectrum of the **5**.

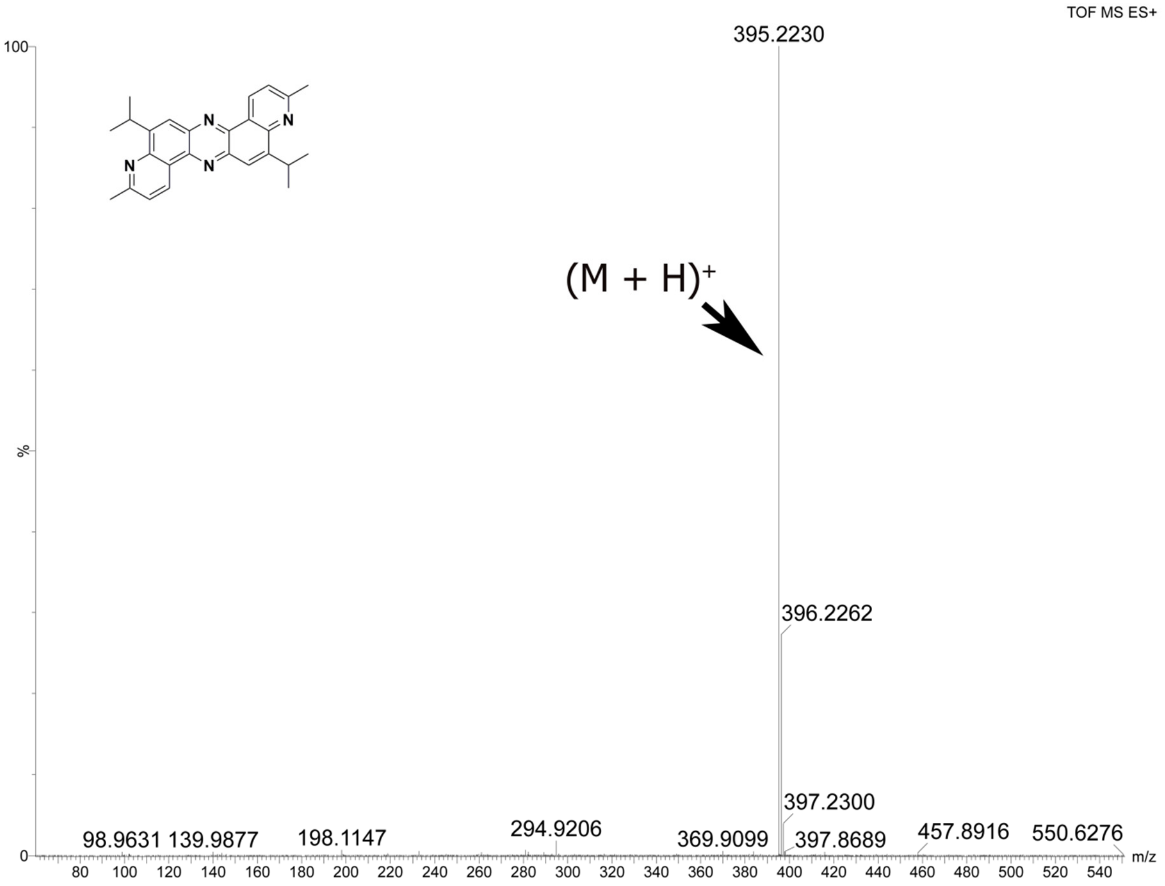

Figure S13c. MS spectrum of the molecule **5**.

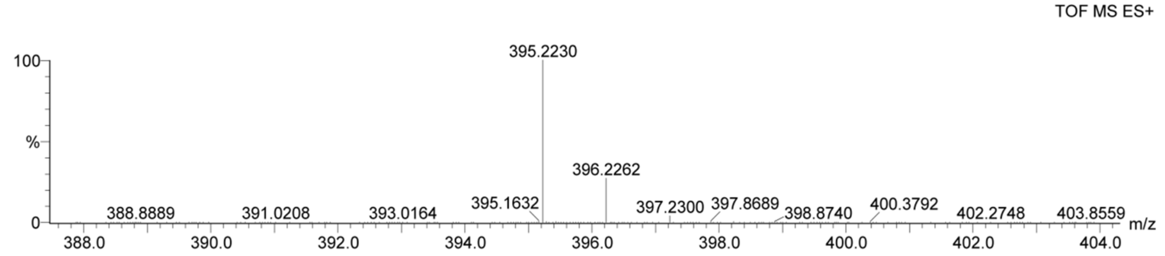

Minimum:				-1.5				
Maximum:		5.0	5.0	80.0				
Mass	Calc. Mass	mDa	PPM	DBE	i-FIT	Norm	Conf(%)	Formula
395.2230	395.2236	-0.6	-1.5	15.5	1024.8	n/a	n/a	C26 H27 N4

Figure S13d. HRMS spectrum of the molecule **5**.

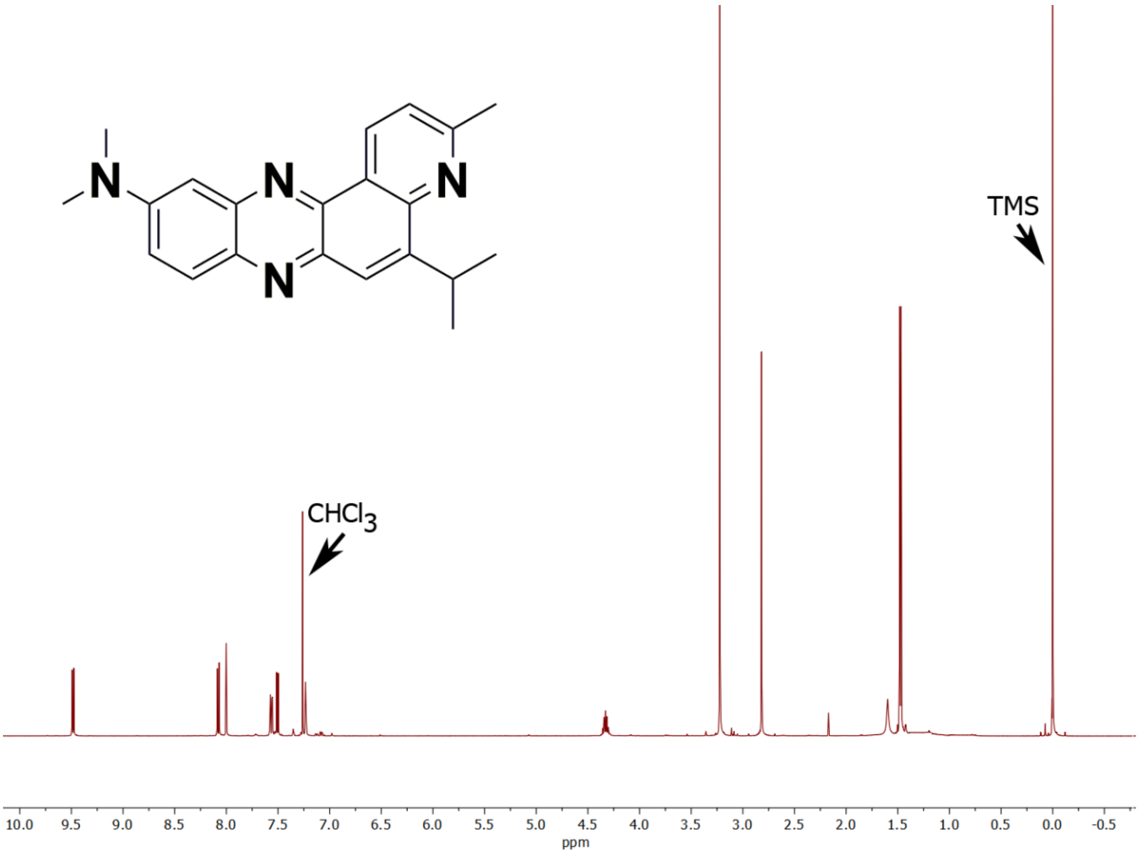

Figure S14a.^{1}H-NMR ($CDCl_3$; 500.2 MHz) spectrum of the **6a**.

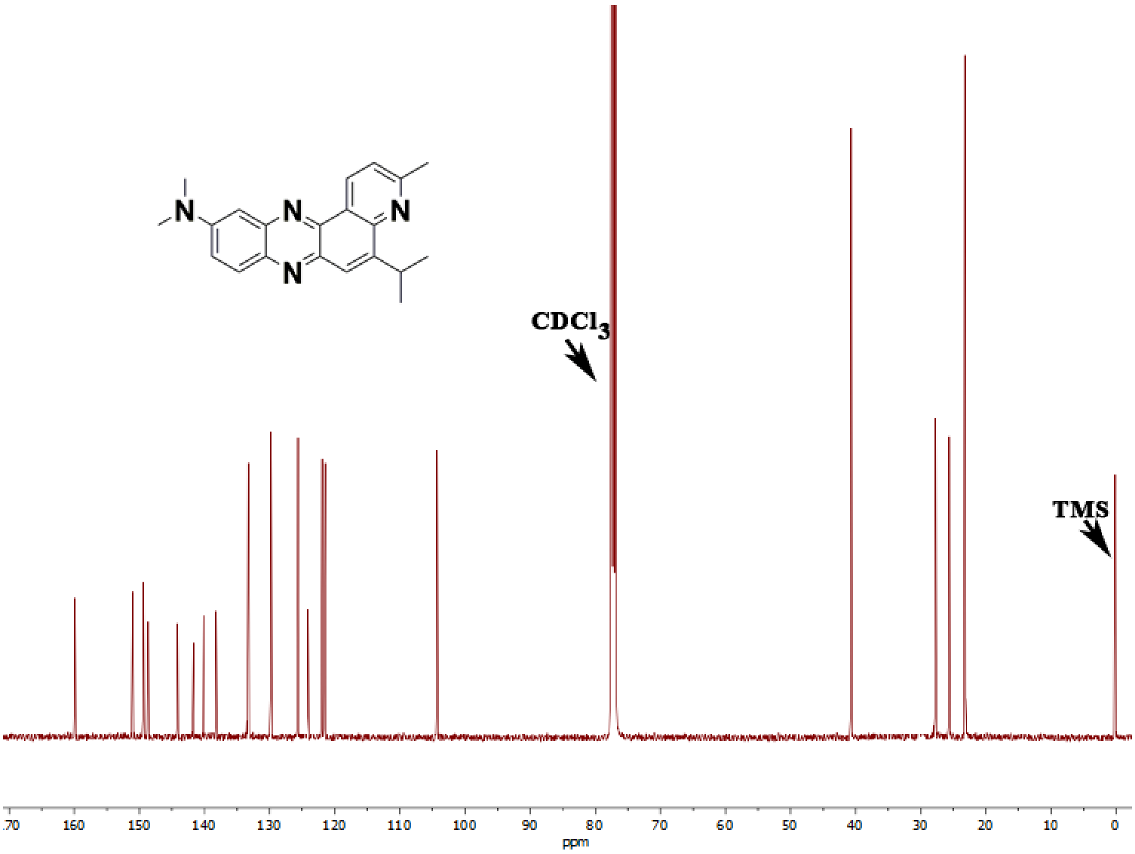

Figure S14b. $^{13}C\{^1H\}$-NMR ($CDCl_3$; 125.8 MHz) spectrum of the **6a**.

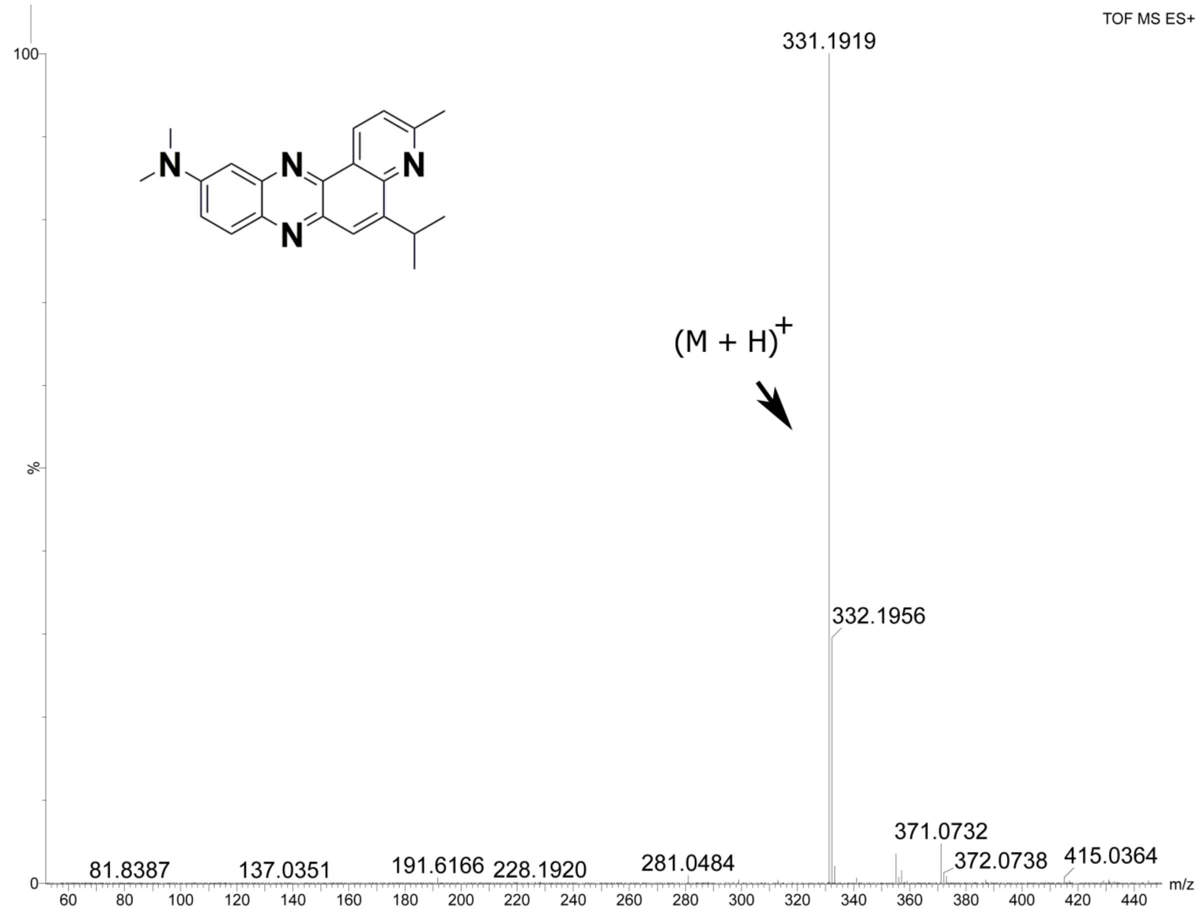

Figure S14c. MS spectrum of the molecule **6a**.

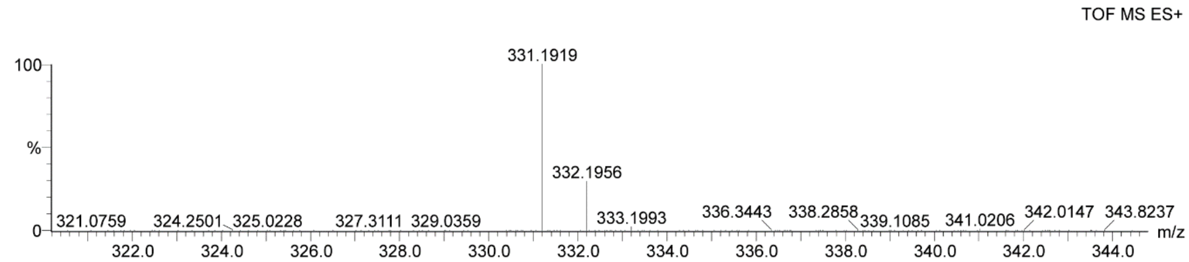

Minimum:				-1.5				
Maximum:		5.0	5.0	90.0				
Mass	Calc. Mass	mDa	PPM	DBE	i-FIT	Norm	Conf(%)	Formula
331.1919	331.1923	-0.4	-1.2	12.5	599.4	n/a	n/a	C21 H23 N4

Figure S14d. HRMS spectrum of the molecule **6a.**

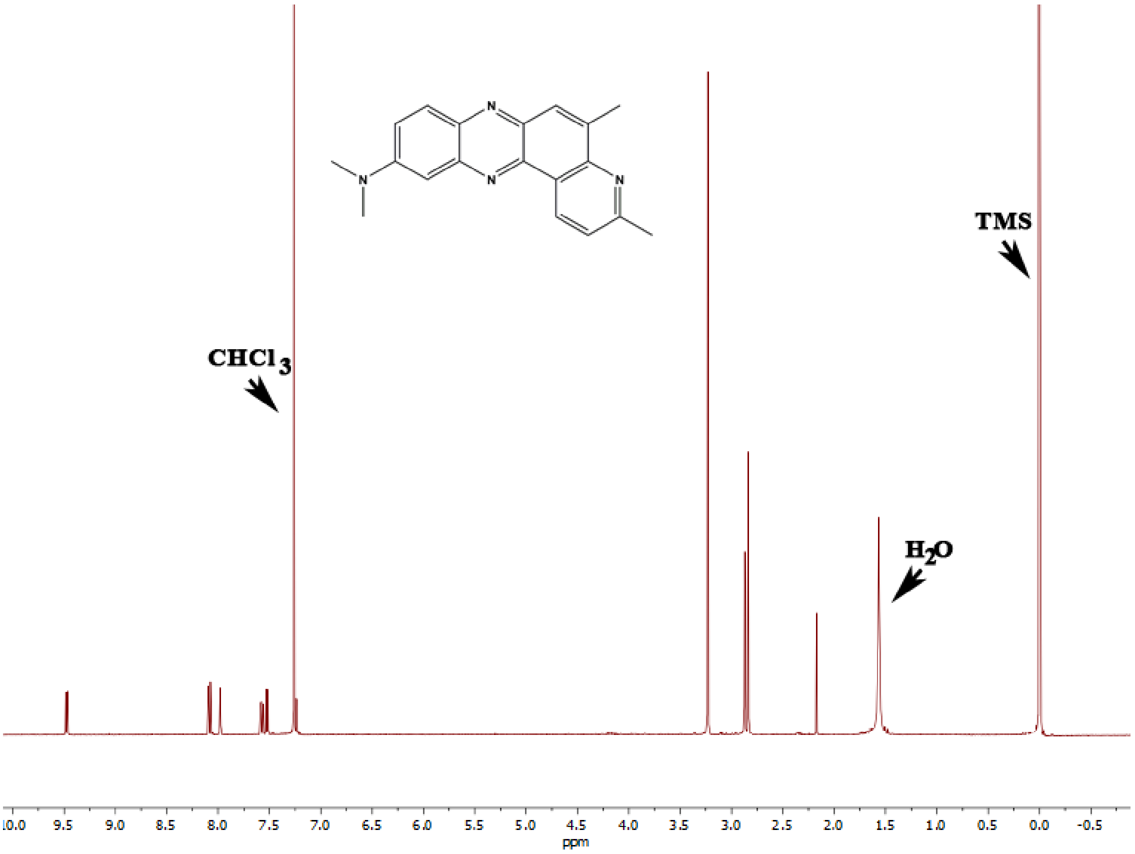

Figure S15a.^{1}H-NMR ($CDCl_3$; 500.2 MHz) spectrum of the **6b**.

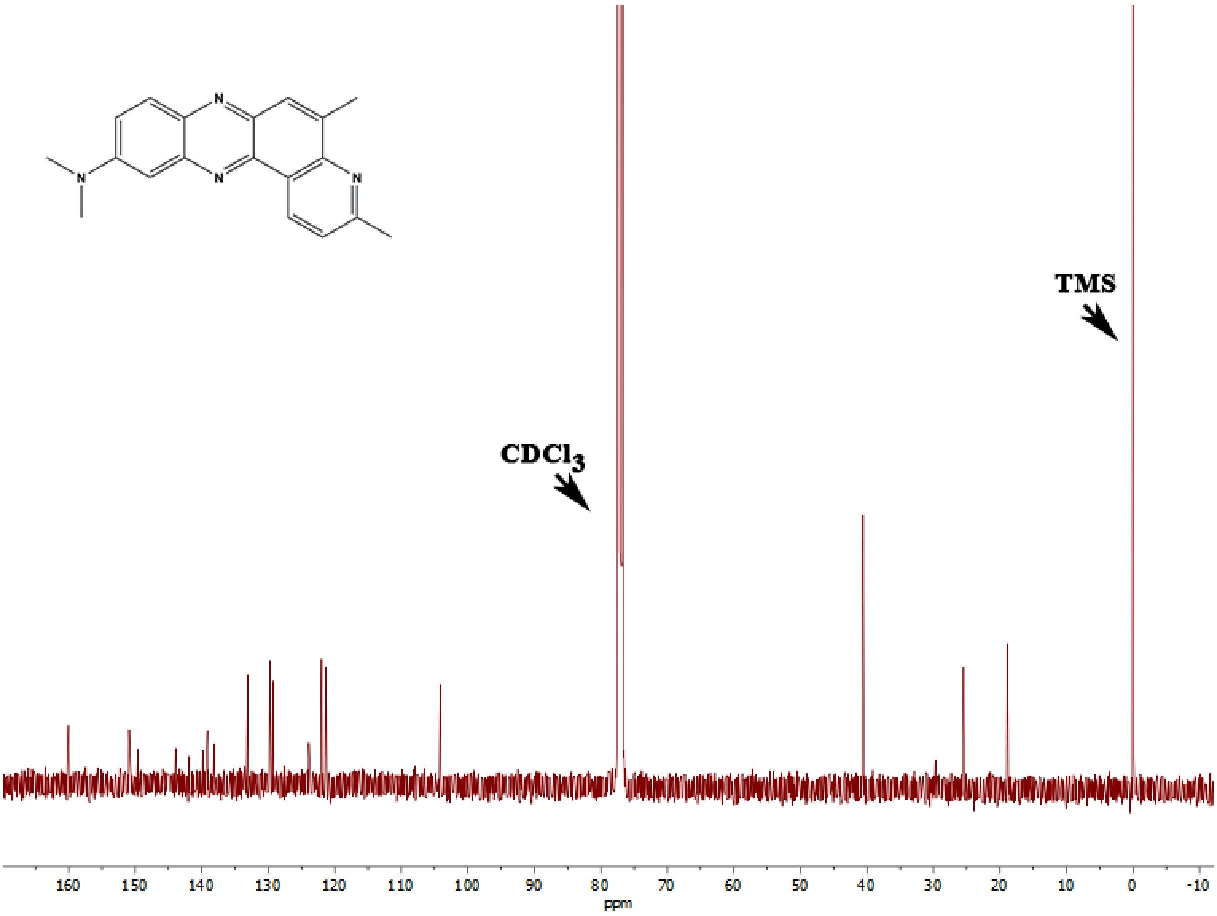

Figure S15b.$^{13}C\{^1H\}$-NMR ($CDCl_3$; 125.8 MHz) spectrum of the **6b**.

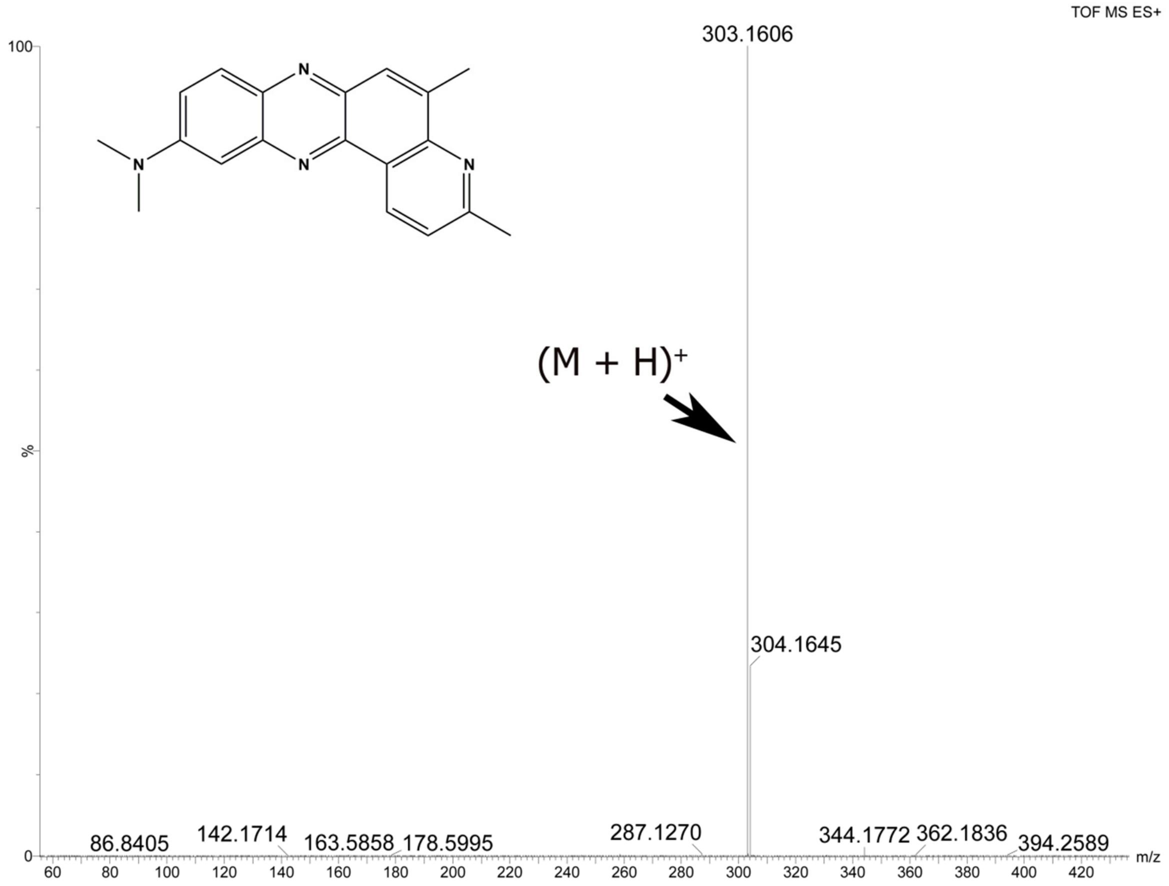

Figure S15c. MS spectrum of the molecule **6b**.

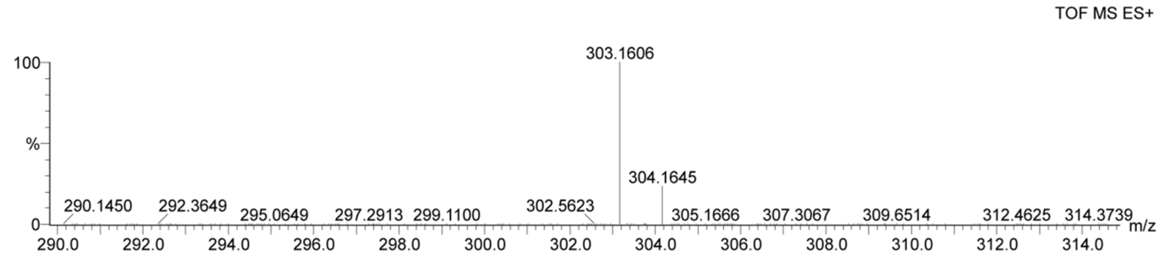

	Mass	Calc. Mass	mDa	PPM	DBE	i-FIT	Norm	Conf(%)	Formula
Minimum:					-1.5				
Maximum:			5.0	5.0	90.0				
	303.1606	303.1610	-0.4	-1.3	12.5	530.4	n/a	n/a	C19 H19 N4

Figure S15d. HRMS spectrum of the molecule **6b**.

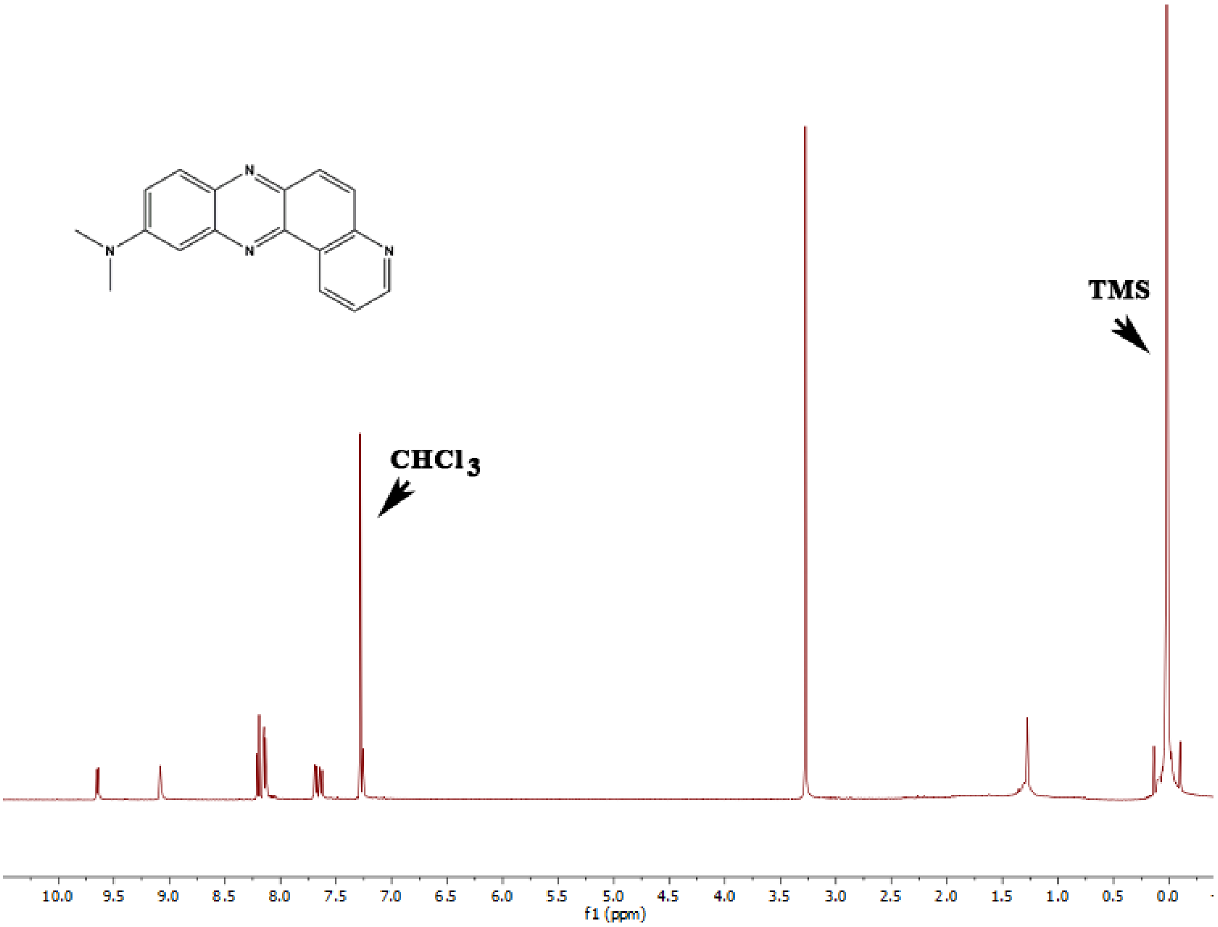

Figure S16a. ^{1}H-NMR ($CDCl_3$; 500.2 MHz) spectrum of the **6c**.

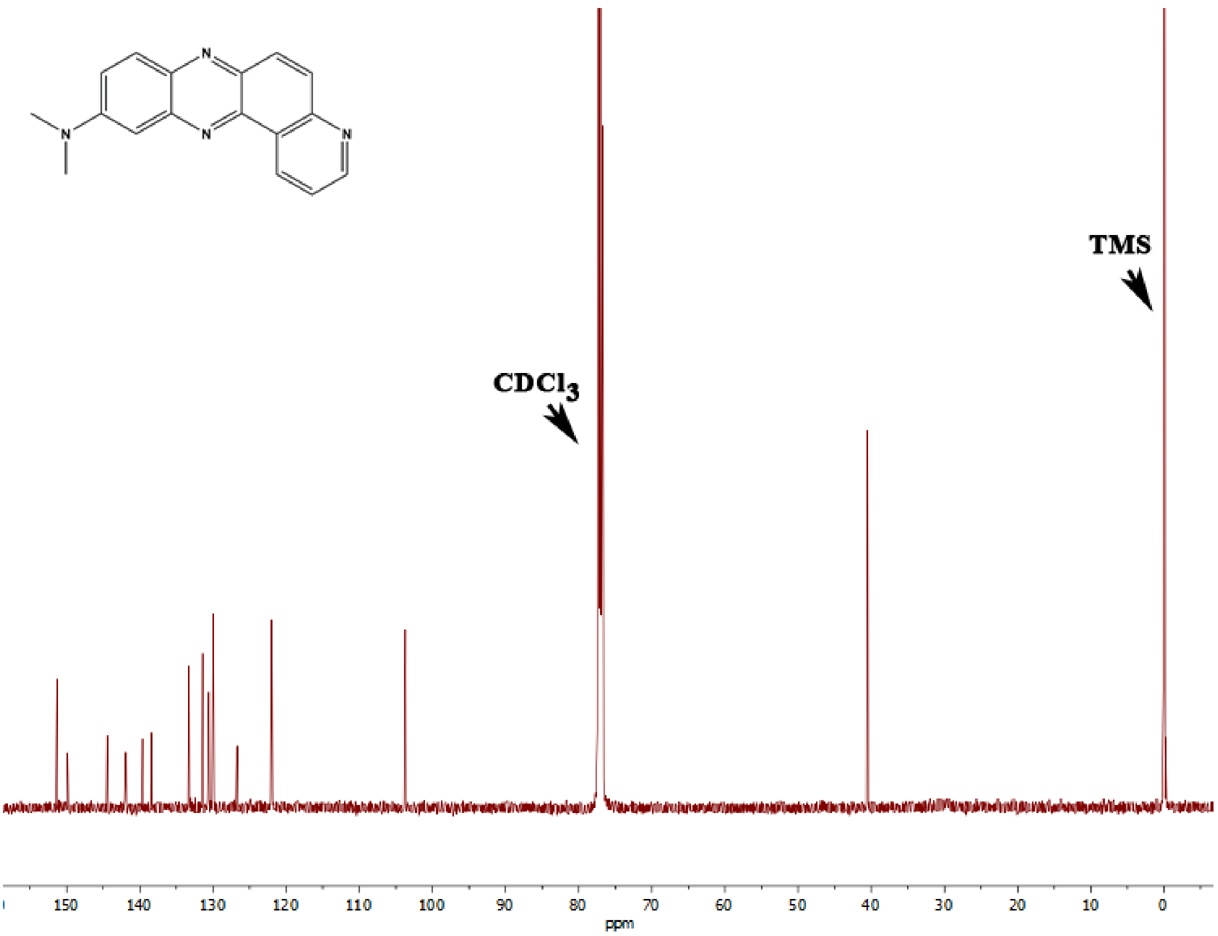

Figure S16b. $^{13}C\{^{1}H\}$-NMR ($CDCl_3$; 125.8 MHz) spectrum of the **6c**.

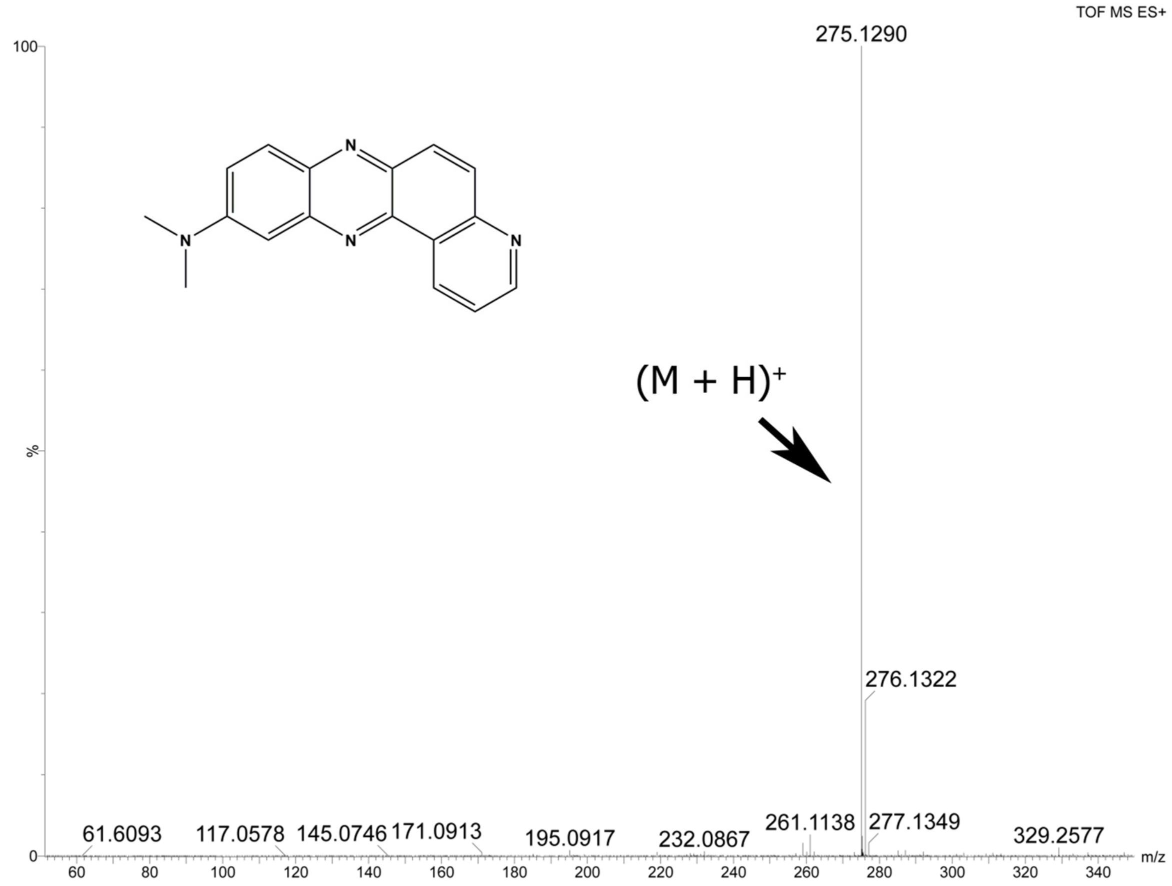

Figure S16c. MS spectrum of the molecule **6c**.

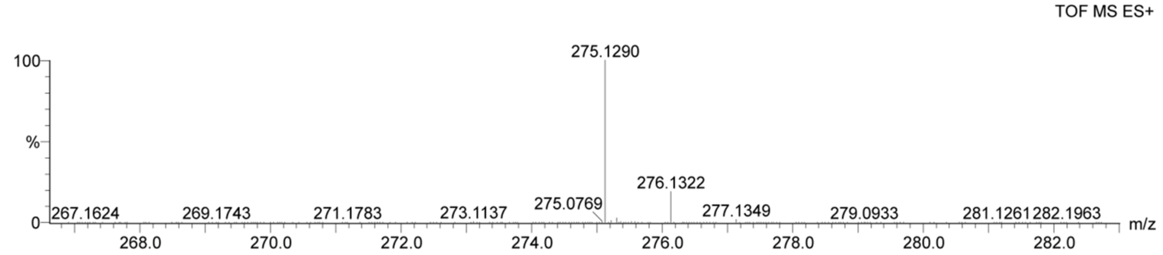

Minimum:				-1.5				
Maximum:		5.0	5.0	80.0				
Mass	Calc. Mass	mDa	PPM	DBE	i-FIT	Norm	Conf(%)	Formula
275.1290	275.1297	-0.7	-2.5	12.5	1034.7	n/a	n/a	C17 H15 N4

Figure S16d. HRMS spectrum of the molecule **6c**.

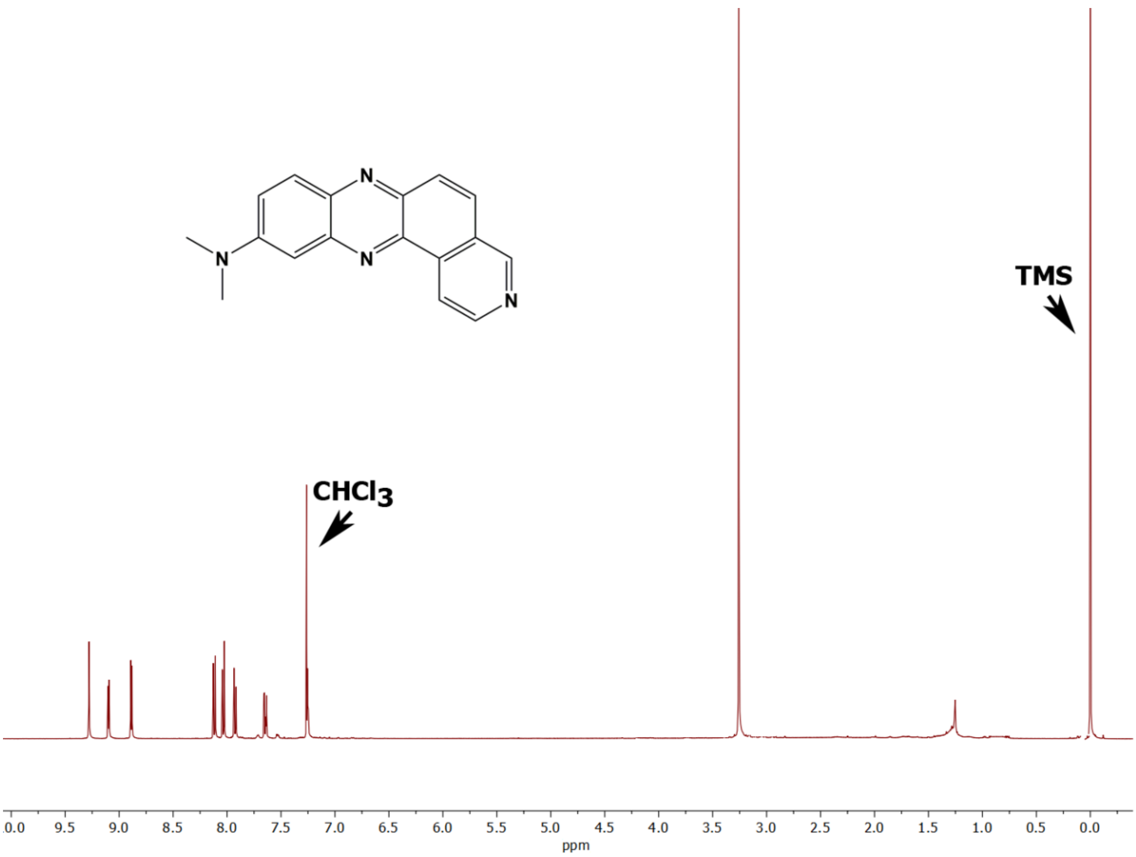

Figure S17a.^{1}H-NMR ($CDCl_3$; 500.2 MHz) spectrum of the **6d**.

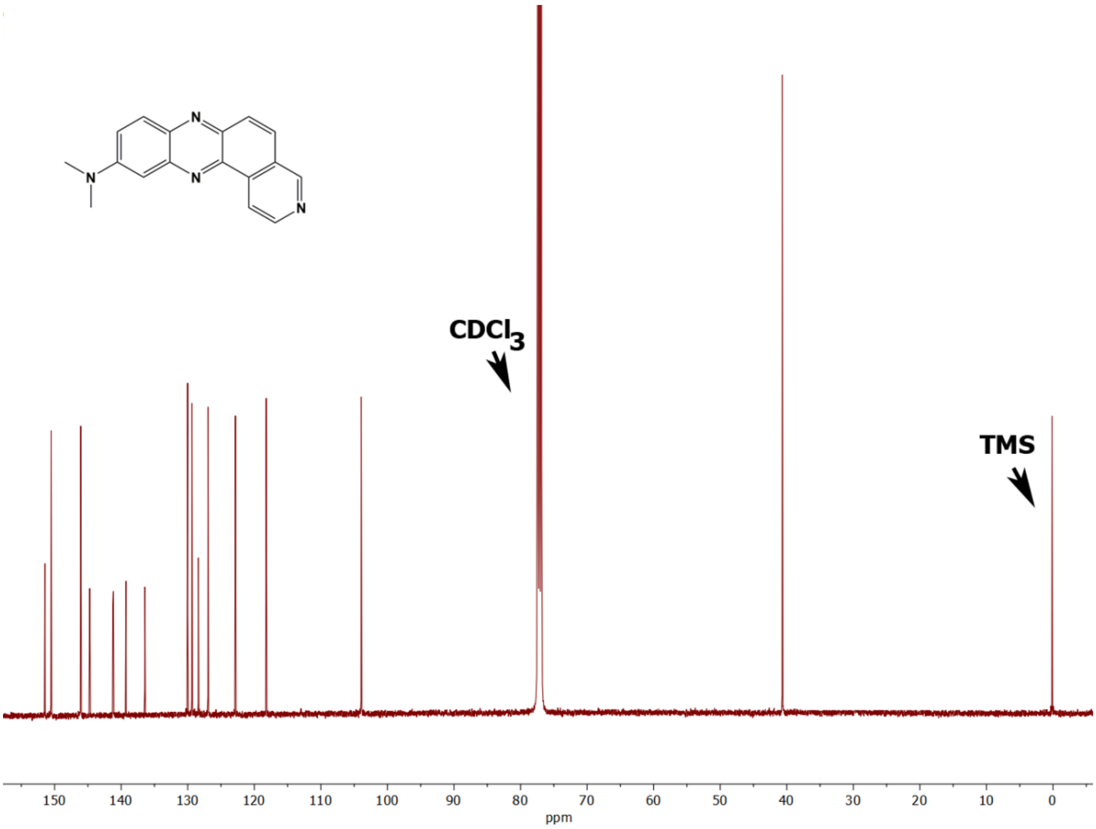

Figure S17b.^{13}C{^{1}H}-NMR ($CDCl_3$; 125.8 MHz) spectrum of the **6d**.

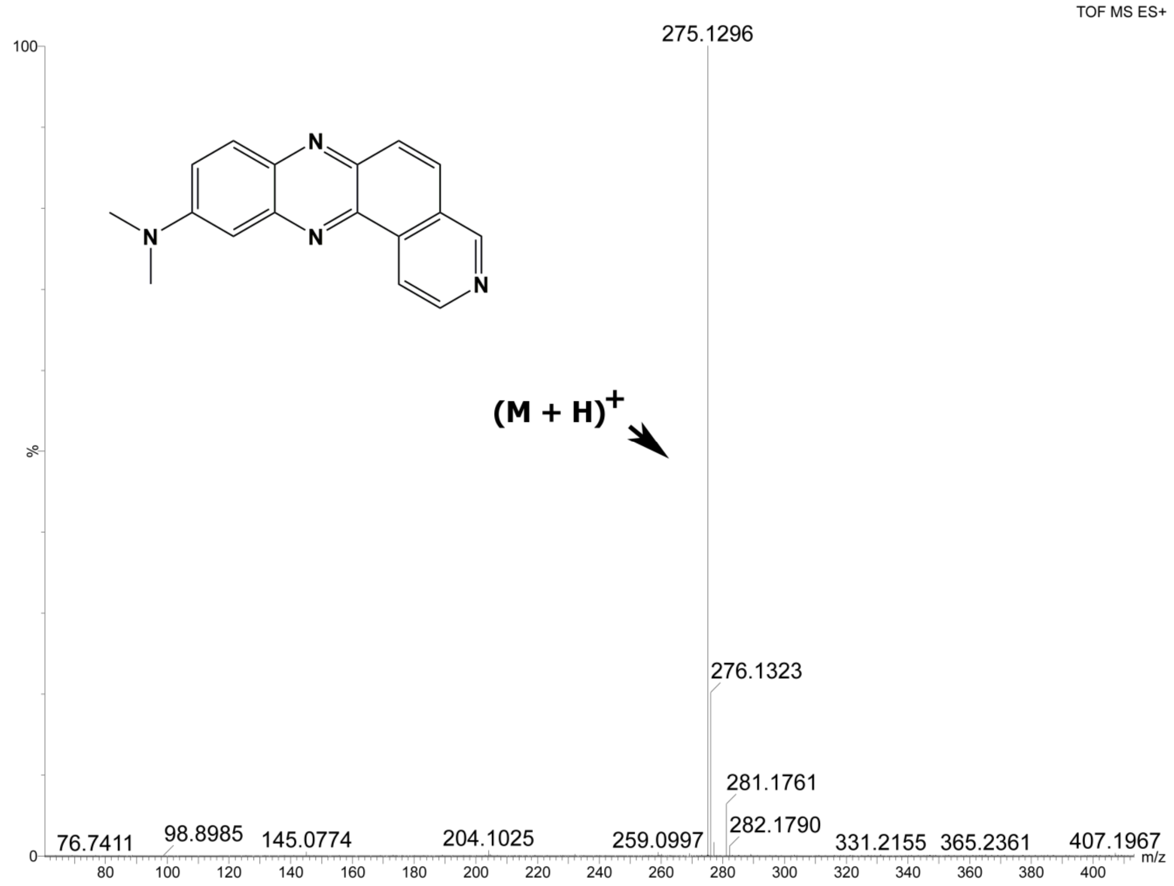

Figure S17c. MS spectrum of the molecule **6d**.

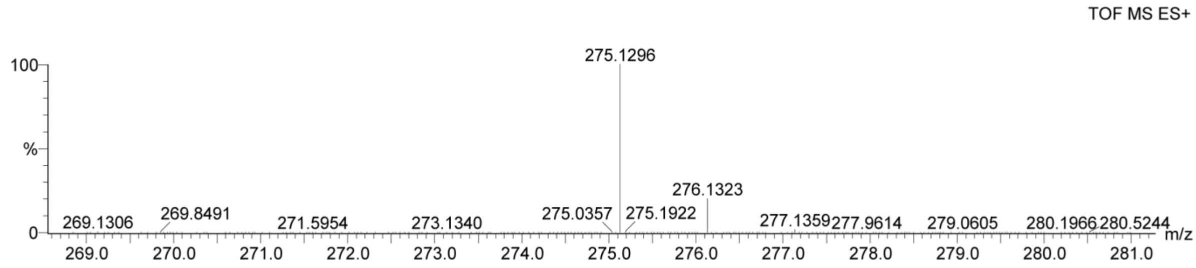

Minimum:				-1.5				
Maximum:		5.0	5.0	90.0				
Mass	Calc. Mass	mDa	PPM	DBE	i-FIT	Norm	Conf(%)	Formula
275.1296	275.1297	-0.1	-0.4	12.5	1143.1	n/a	n/a	C17 H15 N4

Figure S17d. HRMS spectrum of the molecule **6d**.

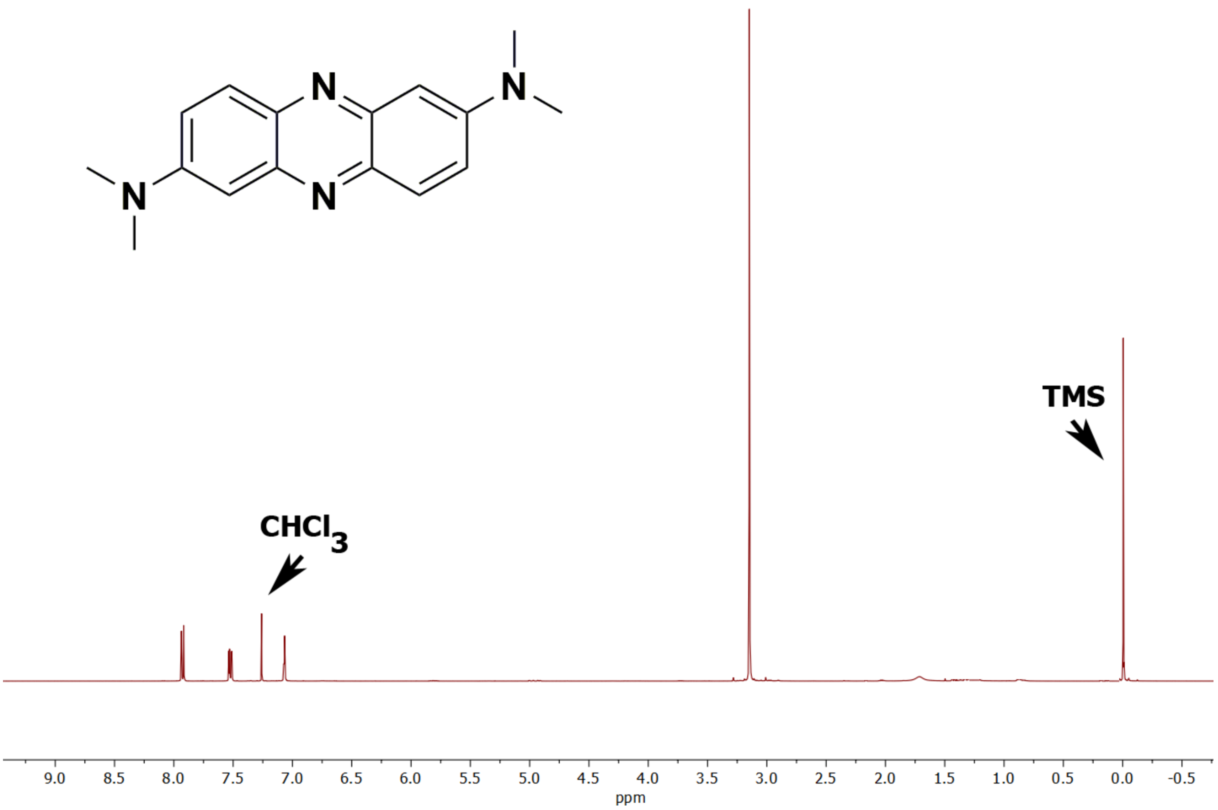

Figure S18a. ^{1}H-NMR ($CDCl_3$; 500.2 MHz) spectrum of the **6e**.

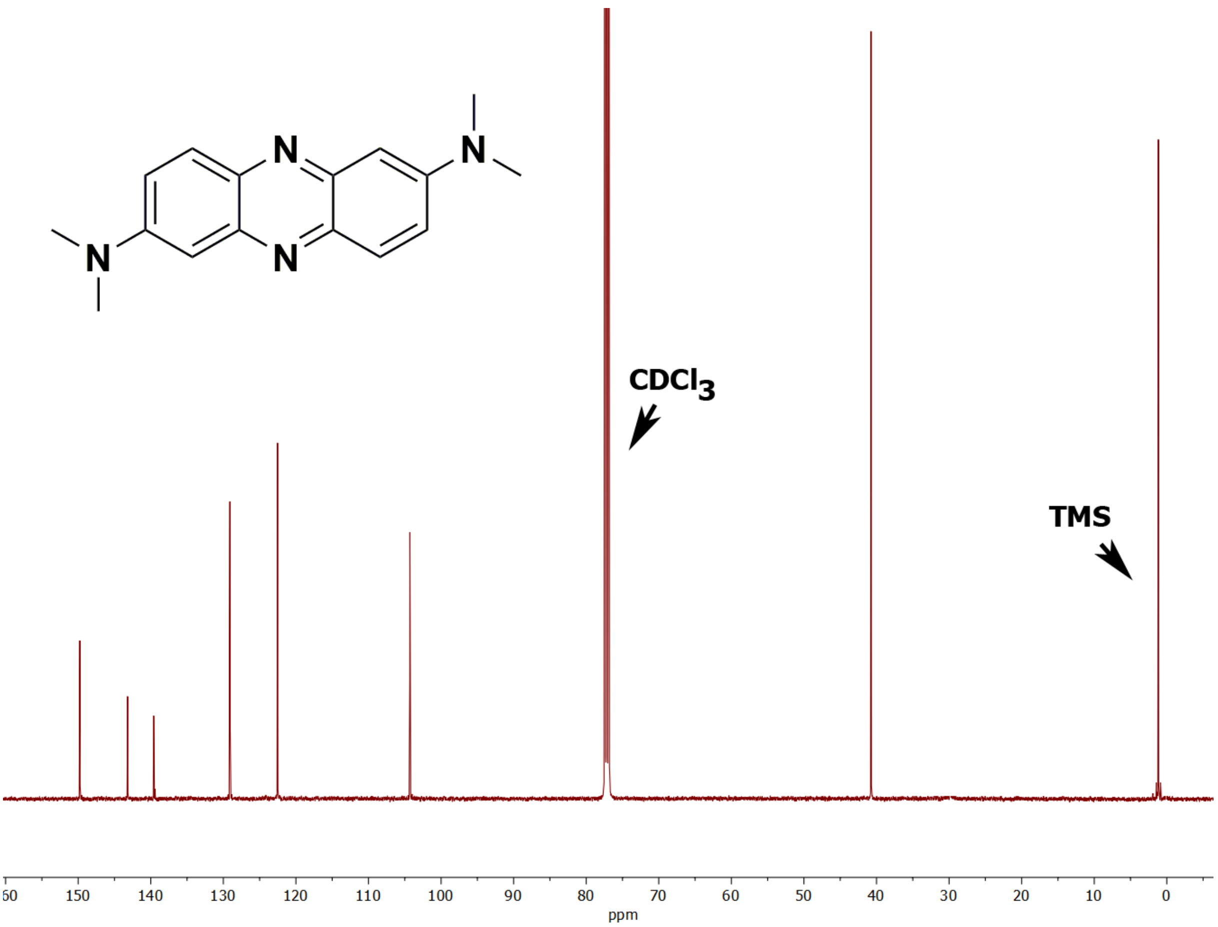

Figure S18b. $^{13}C\{^1H\}$-NMR ($CDCl_3$; 125.8 MHz) spectrum of the **6e**.

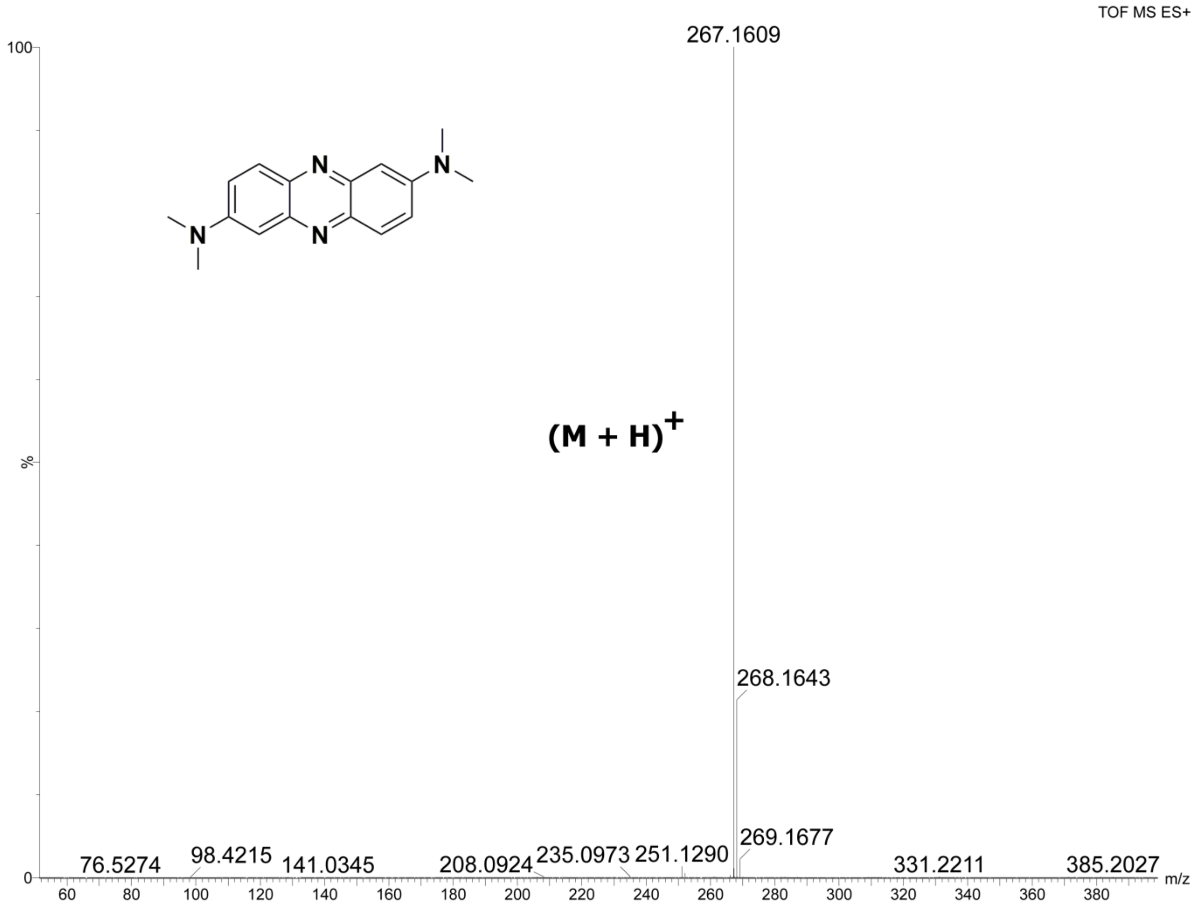

Figure S18c. MS spectrum of the molecule **6e**.

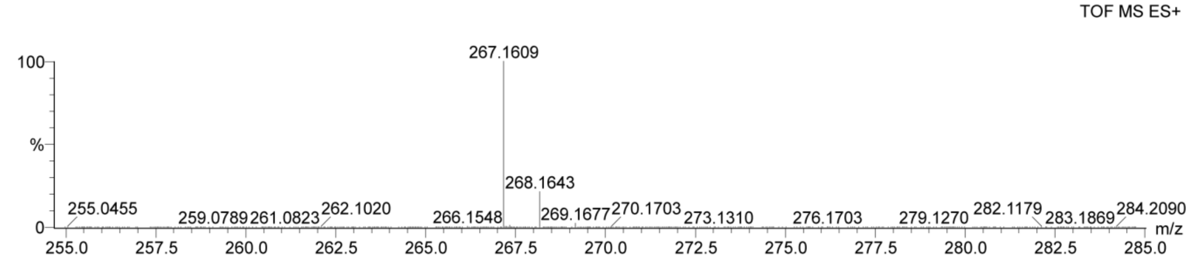

Minimum:				-1.5				
Maximum:		5.0	5.0	90.0				
Mass	Calc. Mass	mDa	PPM	DBE	i-FIT	Norm	Conf(%)	Formula
267.1609	267.1610	-0.1	-0.4	9.5	1476.6	n/a	n/a	C16 H19 N4

Figure S18d. HRMS spectrum of the molecule **6e**.

Table S1. Wavelengths (λ), oscillator strengths (*f*) and orbital assignment of the selected electronic transitions in the absorption spectra of molecules **2a** and **3** calculated at the TDDFT/B3LYP/6-31G(d,p) level of theory

Compounds	Transition	λ, nm	*f*	Assignment
**2a**	$S_0 \to T_3$	306	0	HOMO->LUMO+1(+85%)<br>HOMO-2->LUMO+2(7%)
	$S_0 \to T_2$	350	0	HOMO->LUMO+3(+39%)<br>HOMO-1->LUMO+1(+19%)<br>HOMO-2->LUMO(13%)<br>HOMO-3->LUMO+2(8%)<br>HOMO-3->LUMO(7%)<br>HOMO-2->LUMO+2(+6%)
	$S_0 \to T_1$	358	0	HOMO->LUMO(+60%)<br>HOMO-1->LUMO+2(+12%)<br>HOMO-2->LUMO+3(12%)<br>HOMO-3->LUMO+1(10%)
	$S_0 \to S_1$	286	0.0148	HOMO→LUMO (97%)
	$S_0 \to S_2$	273	0.0141	HOMO→LUMO+1 (90%)
	$S_0 \to S_3$	265	0.0401	HOMO→LUMO+2 (85%)<br>HOMO-1→LUMO (12%)
	$S_0 \to S_4$	250	0.0669	HOMO→ LUMO+3 (90%)
	$S_0 \to S_5$	239	0.2855	HOMO-1→LUMO (70%)<br>HOMO→ LUMO+2 (11%)<br>HOMO→ LUMO+4 (8%)<br>HOMO-2→ LUMO+1 (5%)
	$S_0 \to S_6$	229	0.0218	HOMO-1→ LUMO+1 (88%)
	$S_0 \to S_7$	228	0.0244	HOMO→ LUMO+4 (86%)<br>HOMO-1→ LUMO (5%)
	$S_0 \to S_8$	223	0.0401	HOMO-1→ LUMO+3 (67%)<br>HOMO-2→ LUMO+2 (16%)
	$S_0 \to S_9$	220	0.0039	HOMO-1→ LUMO+2(79%)<br>HOMO-2→ LUMO+1 (13%)
	$S_0 \to S_{10}$	214	0.0067	HOMO-3→ LUMO (38%)<br>HOMO-2→ LUMO (26%)<br>HOMO-1→ LUMO+3 (16%)<br>HOMO-2→ LUMO+2 (10%)<br>HOMO-3→ LUMO+2 (8%)
	$S_0 \to S_{11}$	212	0.1144	HOMO-2→ LUMO+1 (45%)<br>HOMO-3→ LUMO+3 (26%)<br>HOMO-1→ LUMO+2 (9%)<br>HOMO-1→ LUMO (7%)
	$S_0 \to S_{12}$	204	0.0549	HOMO-2→ LUMO (42%)<br>HOMO-3→ LUMO (30%)<br>HOMO-2→ LUMO+2 (12%)<br>HOMO-3→ LUMO+2 (10%)
	$S_0 \to S_{13}$	203	0.0004	HOMO-2→ LUMO+3 (48%)<br>HOMO-3→ LUMO+1 (43%)
	$S_0 \to S_{14}$	196	0.0027	HOMO-1→ LUMO+4 (84%)<br>HOMO-2→ LUMO+3 (9%)

	$S_0 \rightarrow S_{15}$	192	0.5225	HOMO-3→ LUMO+3 (56%)<br>HOMO-2→ LUMO+1 (18%)<br>HOMO-4→ LUMO (7%)<br>HOMO-6→ LUMO (6%)
**3**	$S_0 \rightarrow S_1$	376	0.6989	HOMO→LUMO (99%)
	$S_0 \rightarrow S_2$	330	0.0227	HOMO → LUMO+1 (96%)
	$S_0 \rightarrow S_3$	318	0.0002	HOMO-4→ LUMO (57%)<br>HOMO-5→ LUMO+1(37%)
	$S_0 \rightarrow S_4$	317	0.0010	HOMO-5→ LUMO (58%)<br>HOMO-4→ LUMO+1 (36%)
	$S_0 \rightarrow S_5$	298	0.0076	HOMO→ LUMO+2 (52%)<br>HOMO-1→ LUMO (42%)
	$S_0 \rightarrow S_6$	284	0.0006	HOMO-7 → LUMO+1 (34%)<br>HOMO-6→ LUMO (33%)<br>HOMO-10→ LUMO (27%)
	$S_0 \rightarrow S_7$	283	0.0003	HOMO-7→ LUMO (51%)<br>HOMO-10→ LUMO+1 (19%)<br>HOMO-6→ LUMO+1 (16%)
	$S_0 \rightarrow S_8$	281	0.0011	HOMO-2 → LUMO (60%)<br>HOMO-1→ LUMO+1 (14%)<br>HOMO→ LUMO+3 (13%)
	$S_0 \rightarrow S_9$	272	0.1395	HOMO-1→ LUMO (+51%)<br>HOMO→ LUMO+2(43%)
	$S_0 \rightarrow S_{10}$	259	0.0611	HOMO-1→ LUMO+1(+69%)<br>HOMO-2→ LUMO (24%)
	$S_0 \rightarrow S_{11}$	257	0.0281	HOMO-3→ LUMO (+78%)<br>HOMO-2→ LUMO+1(15%)
	$S_0 \rightarrow S_{12}$	256	0.0047	HOMO → LUMO+3(+78%)<br>HOMO-1→ LUMO+1(+14%)<br>HOMO-2→ LUMO (+5%)
	$S_0 \rightarrow S_{13}$	250	0.0206	HOMO-2→ LUMO+1(+79%)<br>HOMO-3→ LUMO (+17%)
	$S_0 \rightarrow S_{14}$	244	0.0009	HOMO-6→ LUMO (+50%)<br>HOMO-10→ LUMO (+34%)<br>HOMO-4→ LUMO (7%)
	$S_0 \rightarrow S_{15}$	240	0.1145	HOMO-3→LUMO+1(+97%)

All of the transitions are of the $X^1A \rightarrow n^1A$ symmetry. The solvent effects on the vertical absorption spectrum of **2a** and **3** were included using the polarized-continuum model (PCM), taking the acetonitrile (MeCN) for **2a** and dimethylsulfoxide for **3** as model solvents.

Table S2. Spectroscopic data of the S_1–S_0 electronic transition in the emission spectra of the studied compounds calculated at the B3LYP/6-31G(d,p) level of theory

Compound	Transition	$\lambda_{em.}$, nm	f
**2a**	S_1–S_0	386	0.0013
**3**	S_1–S_0	568	0.0201

Table S3. Crystal data and structure refinement details of compounds **1, 2a**, **2b**, **3, 5** and **6a**.

	**1**	**2a**	**2b**	**3**	**5**	**6a**
Empirical formula	$C_{21}H_{13}N_3OS$	$C_{17}H_{18}NO_3PS$	$C_5H_{11}O_3PS$	$C_{13}H_{11}N_3O_6S$	$C_{26}H_{26}N_4$	$C_{21}H_{22}N_4$
Temperature (K)	292	294	294	100	294	100
Wavelength (Å)	0.71073	1.54184	0.71073	0.71073	0.71073	0.71073
Crystal system	Monoclinic	Monoclinic	Orthorhombic	Monoclinic	Tetragonal	Triclinic
Space group	$P2_1$/n	$P2_1$/n	*P*bca	$P2_1$/n	$P4_2$/n	*P*-1
Unit cell dimensions						
a (Å)	12.7017(6)	8.8248(3)	11.1587(17)	8.2753(3)	21.1688(8)	9.0731(6)
b (Å)	8.8798(4)	16.5758(4)	11.8846(13)	19.9331(7)	21.1688(8)	9.3169(7)
c (Å)	15.0016(6)	11.8216(4)	13.1114(16)	8.5875(3)	4.6776(3)	11.5962(8)
α (°)	90	90	90	90	90	91.441(6)
β (°)	103.353(5)	103.951(3)	90	99.197(3)	90	104.105(5)
γ (°)	90	90	90	90	90	116.266(7)
Volume ($Å^3$)	1646.27(13)	1678.23(9)	1738.8(4)	1398.32(9)	2096.1(2)	842.52(11)
Z	4	4	8	4	4	2
Calculated density(g/cm^3)	1.434	1.375	1.392	1.602	1.250	1.302
Absorption coefficient (mm^{-1})	0.212	2.735	0.508	0.270	0.075	0.079
F(000)	736	728	768	696	840	352
Crystal dimensions (mm)	0.28 x 0.09 x 0.08	0.53 x 0.11 x 0.08	0.41 x 0.07 x 0.05	0.64 x 0.08 x 0.08	0.42 x 0.04 x 0.04	0.53 x 0.15 x 0.05
θ range for data collection (°)	3.297 to 26.372	4.687 to 73.641	3.428 to 26.368	3.155 to 26.364	3.043 to 26.347	3.291 to 26.369
Index ranges	$-11 \leq h \leq 15$<br>$-11 \leq k \leq 11$<br>$-18 \leq l \leq 18$	$-8 \leq h \leq 10$<br>$-20 \leq k \leq 20$<br>$-14 \leq l \leq 14$	$-10 \leq h \leq 13$<br>$-14 \leq k \leq 12$<br>$-16 \leq l \leq 15$	$-10 \leq h \leq 10$<br>$-24 \leq k \leq 24$<br>$-9 \leq l \leq 10$	$-26 \leq h \leq 26$<br>$-60 \leq k \leq 24$<br>$-5 \leq l \leq 5$	$-11 \leq h \leq 11$<br>$-8 \leq k \leq 11$<br>$-14 \leq l \leq 14$
Reflections collected	13281	11350	8570	11655	16533	7141
Independent reflections	3354	3338	1772	2859	2137	3450
Data / restraints / parameters	3354 / 0 / 235	3338 / 0 / 211	1772 / 2 / 106	2859 / 5 / 227	2137 / 0 / 139	3450 / 0 / 231
Goodness-of-fit on F^2	1.032	1.059	1.000	1.110	1.042	1.022
Final R indices ($I>2\sigma(I)$)	0.0353; 0.0933	0.0462; 0.1268	0.0591; 0.1458	0.0602; 0.1847	0.0555; 0.1476	0.0468; 0.1091
R indices (all data)	0.0452; 0.1022	0.0520; 0.1348	0.1116; 0.1812	0.0692; 0.1930	0.0878; 0.1757	0.0726; 0.1290
Largest diff. peak and hole	0.197 and -0.191	0.278 and -0.370	0.322 and -0.281	1.233 and -0.551	0.198 and -0.158	0.217 and -0.304
CCDC number	2177507	2177508	2178722	2177509	2177510	2177511

Table S4. Geometric parameters (Å, º) for 1

S11—C11A	1.7452 (16)	C4A—C12A	1.402 (2)
S11—C10A	1.7494 (17)	C5A—C11A	1.421 (2)
O1—C13	1.1991 (18)	C6A—C7	1.376 (2)
N5—C5A	1.2905 (19)	C6A—C10A	1.384 (2)
N5—C4A	1.3612 (18)	C7—C8	1.373 (2)

N6—C13	1.3935 (18)	C8—C9	1.366 (3)
N6—C5A	1.4028 (18)	C9—C10	1.364 (3)
N6—C6A	1.4236 (18)	C10—C10A	1.377 (2)
N12—C11A	1.2947 (19)	C13—C14	1.480 (2)
N12—C12A	1.361 (2)	C14—C19	1.377 (2)
C1—C2	1.351 (3)	C14—C15	1.379 (2)
C1—C12A	1.401 (2)	C15—C16	1.374 (3)
C2—C3	1.384 (3)	C16—C17	1.360 (3)
C3—C4	1.355 (2)	C17—C18	1.368 (3)
C4—C4A	1.394 (2)	C18—C19	1.373 (2)
C11A—S11—C10A	97.81 (7)	C9—C10—C10A	119.71 (16)
C5A—N5—C4A	116.57 (12)	C10—C10A—C6A	120.15 (16)
C13—N6—C5A	122.14 (12)	C10—C10A—S11	119.46 (13)
C13—N6—C6A	121.67 (12)	C6A—C10A—S11	120.38 (12)
C5A—N6—C6A	116.07 (11)	N12—C11A—C5A	122.25 (14)
C11A—N12—C12A	116.35 (13)	N12—C11A—S11	118.72 (11)
C2—C1—C12A	119.65 (17)	C5A—C11A—S11	118.98 (11)
C1—C2—C3	121.04 (16)	N12—C12A—C1	119.55 (15)
C4—C3—C2	120.92 (18)	N12—C12A—C4A	121.36 (13)
C3—C4—C4A	119.44 (17)	C1—C12A—C4A	119.10 (15)
N5—C4A—C4	119.38 (14)	O1—C13—N6	121.17 (15)
N5—C4A—C12A	120.77 (14)	O1—C13—C14	121.96 (14)
C4—C4A—C12A	119.84 (14)	N6—C13—C14	116.78 (13)
N5—C5A—N6	119.03 (12)	C19—C14—C15	119.54 (16)
N5—C5A—C11A	122.63 (13)	C19—C14—C13	121.63 (14)
N6—C5A—C11A	118.34 (13)	C15—C14—C13	118.61 (16)
C7—C6A—C10A	119.54 (14)	C16—C15—C14	119.53 (19)
C7—C6A—N6	122.49 (13)	C17—C16—C15	120.57 (18)
C10A—C6A—N6	117.92 (13)	C16—C17—C18	120.27 (19)
C8—C7—C6A	119.63 (16)	C17—C18—C19	119.8 (2)
C9—C8—C7	120.59 (17)	C18—C19—C14	120.28 (17)
C10—C9—C8	120.36 (17)		

Table S5. Geometric parameters (Å, º) for 2a

P1—O3	1.4523 (17)	C4A—C9A	1.391 (3)
P1—O1	1.5695 (14)	C4A—N10	1.430 (2)
P1—O2	1.5705 (15)	C5—C10A	1.387 (3)
P1—N10	1.6697 (15)	C5—C6	1.395 (3)

S9—C8A	1.764 (3)	C6—C7	1.368 (5)
S9—C9A	1.764 (3)	C7—C8	1.377 (4)
O1—C11	1.451 (3)	C8—C8A	1.391 (3)
O2—C13	1.452 (3)	C8A—C10A	1.388 (3)
C1—C2	1.373 (4)	N10—C10A	1.433 (2)
C1—C9A	1.390 (3)	C11—C12	1.521 (3)
C2—C3	1.362 (4)	C12—C14	1.521 (3)
C3—C4	1.388 (3)	C12—C13	1.525 (3)
C4—C4A	1.389 (3)	C12—C15	1.529 (3)
O3—P1—O1	112.36 (10)	C10A—C8A—C8	120.0 (2)
O3—P1—O2	112.66 (10)	C10A—C8A—S9	118.61 (16)
O1—P1—O2	106.36 (8)	C8—C8A—S9	121.4 (2)
O3—P1—N10	117.01 (10)	C1—C9A—C4A	119.6 (2)
O1—P1—N10	103.66 (7)	C1—C9A—S9	122.1 (2)
O2—P1—N10	103.75 (8)	C4A—C9A—S9	118.23 (16)
C8A—S9—C9A	97.89 (10)	C4A—N10—C10A	115.59 (14)
C11—O1—P1	118.43 (11)	C4A—N10—P1	122.18 (12)
C13—O2—P1	117.54 (12)	C10A—N10—P1	120.66 (12)
C2—C1—C9A	120.2 (3)	C5—C10A—C8A	119.79 (19)
C3—C2—C1	120.5 (2)	C5—C10A—N10	121.93 (18)
C2—C3—C4	120.4 (3)	C8A—C10A—N10	118.22 (18)
C3—C4—C4A	119.9 (2)	O1—C11—C12	112.17 (15)
C4—C4A—C9A	119.38 (19)	C11—C12—C14	108.24 (19)
C4—C4A—N10	122.00 (18)	C11—C12—C13	108.12 (16)
C9A—C4A—N10	118.57 (18)	C14—C12—C13	109.10 (18)
C10A—C5—C6	119.3 (2)	C11—C12—C15	110.45 (16)
C7—C6—C5	120.8 (3)	C14—C12—C15	110.02 (19)
C6—C7—C8	120.1 (2)	C13—C12—C15	110.83 (17)
C7—C8—C8A	120.0 (3)	O2—C13—C12	112.00 (16)

Table S6. Geometric parameters (Å, º) for 2b.

P1—O1	1.446 (4)	C3—C4	1.527 (6)
P1—O2	1.544 (3)	C4—C5	1.512 (5)
P1—O3	1.551 (3)	C4—C9	1.527 (6)
P1—S1B	2.0006 (10)	C4—C10	1.529 (6)
P1—S1A	2.0006 (10)	C5—O3	1.471 (5)
O2—C3	1.470 (5)		

O1—P1—O2	114.5 (2)	O2—C3—C4	111.1 (3)
O1—P1—O3	114.4 (2)	C5—C4—C3	108.3 (3)
O2—P1—O3	106.97 (17)	C5—C4—C9	107.1 (3)
O1—P1—S1B	112.7 (5)	C3—C4—C9	108.7 (4)
O2—P1—S1B	106.6 (7)	C5—C4—C10	111.5 (3)
O3—P1—S1B	100.5 (7)	C3—C4—C10	110.4 (4)
O1—P1—S1A	110.5 (6)	C9—C4—C10	110.7 (4)
O2—P1—S1A	101.0 (4)	O3—C5—C4	111.8 (3)
O3—P1—S1A	108.5 (5)	C5—O3—P1	119.0 (2)
C3—O2—P1	119.4 (2)		

Table S7. Geometric parameters (Å, º) for 3.

C99—O99	1.4489 (10)	N10—C4A	1.368 (4)
O98—C98	1.4496 (10)	C1—C2	1.377 (4)
O96—C96	1.4489 (10)	C1—C9A	1.390 (4)
S9—O5A	1.497 (3)	C2—C3	1.385 (5)
S9—O5B	1.497 (5)	C3—C4	1.372 (5)
S9—C9A	1.765 (3)	C4—C4A	1.407 (4)
S9—C8A	1.769 (3)	C4A—C9A	1.408 (4)
N1—O2	1.227 (4)	C5—C6	1.372 (5)
N1—O1	1.233 (4)	C5—C10A	1.412 (4)
N1—C2	1.461 (4)	C6—C7	1.388 (5)
N2—O4	1.218 (4)	C7—C8	1.384 (4)
N2—O3	1.229 (4)	C8—C8A	1.391 (4)
N2—C7	1.453 (4)	C8A—C10A	1.403 (4)
N10—C10A	1.363 (4)		
O5A—S9—C9A	105.78 (14)	N10—C4A—C4	118.6 (3)
O5B—S9—C9A	108.4 (17)	N10—C4A—C9A	122.7 (3)
O5A—S9—C8A	105.69 (14)	C4—C4A—C9A	118.7 (3)
O5B—S9—C8A	109.5 (17)	C6—C5—C10A	120.8 (3)
C9A—S9—C8A	98.32 (15)	C5—C6—C7	119.1 (3)
O2—N1—O1	123.7 (3)	C8—C7—C6	122.2 (3)
O2—N1—C2	119.1 (3)	C8—C7—N2	119.0 (3)
O1—N1—C2	117.2 (3)	C6—C7—N2	118.8 (3)
O4—N2—O3	123.6 (3)	C7—C8—C8A	118.5 (3)
O4—N2—C7	118.4 (3)	C8—C8A—C10A	120.8 (3)
O3—N2—C7	118.0 (3)	C8—C8A—S9	115.3 (2)
C10A—N10—C4A	124.5 (3)	C10A—C8A—S9	123.3 (2)

C2—C1—C9A	118.5 (3)	C1—C9A—C4A	120.5 (3)
C1—C2—C3	122.5 (3)	C1—C9A—S9	115.3 (2)
C1—C2—N1	118.1 (3)	C4A—C9A—S9	123.4 (2)
C3—C2—N1	119.3 (3)	N10—C10A—C8A	123.1 (3)
C4—C3—C2	119.0 (3)	N10—C10A—C5	118.3 (3)
C3—C4—C4A	120.8 (3)	C8A—C10A—C5	118.6 (3)

Table S8. Geometric parameters (Å, º) for 5.

N1—C2	1.338 (2)	C5—C13	1.508 (3)
N1—C3^{i}	1.344 (2)	C6—C7	1.399 (3)
N11—C10	1.333 (3)	C7—C8	1.399 (3)
N11—C6	1.361 (2)	C8—C9	1.367 (3)
C2—C3	1.417 (3)	C9—C10	1.394 (3)
C2—C7	1.446 (3)	C10—C12	1.506 (3)
C3—C4	1.424 (3)	C13—C15	1.520 (3)
C4—C5	1.346 (3)	C13—C14	1.524 (3)
C5—C6	1.463 (3)		
C2—N1—C3^{i}	116.67 (17)	N11—C6—C5	117.68 (18)
C10—N11—C6	118.11 (18)	C7—C6—C5	120.12 (18)
N1—C2—C3	121.72 (18)	C8—C7—C6	118.14 (18)
N1—C2—C7	119.85 (17)	C8—C7—C2	121.64 (19)
C3—C2—C7	118.43 (18)	C6—C7—C2	120.22 (17)
N1^{i}—C3—C2	121.61 (18)	C9—C8—C7	119.4 (2)
N1^{i}—C3—C4	118.90 (18)	C8—C9—C10	119.3 (2)
C2—C3—C4	119.48 (18)	N11—C10—C9	122.83 (19)
C5—C4—C3	123.27 (18)	N11—C10—C12	116.5 (2)
C4—C5—C6	118.40 (19)	C9—C10—C12	120.7 (2)
C4—C5—C13	122.96 (19)	C5—C13—C15	110.08 (19)
C6—C5—C13	118.62 (18)	C5—C13—C14	113.83 (19)
N11—C6—C7	122.19 (18)	C15—C13—C14	110.8 (2)

Symmetrycode(s): (i) -*x*, -*y*, -*z*+1.

Table S9. Geometric parameters (Å, º) for 6a.

N4—C3	1.3331 (18)	C4A—C5	1.4618 (19)
N4—C4A	1.3576 (19)	C5—C6	1.349 (2)
N7—C6A	1.3387 (19)	C5—C14	1.5161 (19)
N7—C7A	1.3418 (18)	C6—C6A	1.433 (2)

N12—C12A	1.3353 (18)	C6A—C12A	1.433 (2)
N12—C11A	1.3555 (19)	C7A—C8	1.417 (2)
N17—C10	1.3719 (19)	C7A—C11A	1.435 (2)
N17—C19	1.447 (2)	C8—C9	1.353 (2)
N17—C18	1.4523 (19)	C9—C10	1.440 (2)
C1—C2	1.365 (2)	C10—C11	1.386 (2)
C1—C12B	1.4045 (19)	C11—C11A	1.408 (2)
C2—C3	1.4038 (19)	C12A—C12B	1.443 (2)
C3—C13	1.496 (2)	C14—C16	1.524 (2)
C4A—C12B	1.4094 (19)	C14—C15	1.527 (2)
C3—N4—C4A	118.50 (12)	N7—C7A—C8	119.81 (13)
C6A—N7—C7A	116.68 (12)	N7—C7A—C11A	122.29 (14)
C12A—N12—C11A	116.86 (13)	C8—C7A—C11A	117.89 (13)
C10—N17—C19	119.44 (14)	C9—C8—C7A	121.20 (14)
C10—N17—C18	121.80 (13)	C8—C9—C10	121.72 (15)
C19—N17—C18	118.75 (13)	N17—C10—C11	122.22 (14)
C2—C1—C12B	119.55 (13)	N17—C10—C9	119.85 (14)
C1—C2—C3	119.56 (13)	C11—C10—C9	117.93 (14)
N4—C3—C2	122.32 (14)	C10—C11—C11A	121.19 (14)
N4—C3—C13	117.75 (12)	N12—C11A—C11	119.42 (13)
C2—C3—C13	119.93 (13)	N12—C11A—C7A	120.57 (13)
N4—C4A—C12B	122.48 (13)	C11—C11A—C7A	120.00 (14)
N4—C4A—C5	117.44 (12)	N12—C12A—C6A	122.11 (14)
C12B—C4A—C5	120.07 (13)	N12—C12A—C12B	119.93 (13)
C6—C5—C4A	118.49 (13)	C6A—C12A—C12B	117.95 (13)
C6—C5—C14	123.32 (13)	C1—C12B—C4A	117.60 (14)
C4A—C5—C14	118.19 (13)	C1—C12B—C12A	121.63 (13)
C5—C6—C6A	123.36 (13)	C4A—C12B—C12A	120.77 (13)
N7—C6A—C12A	121.45 (13)	C5—C14—C16	113.27 (13)
N7—C6A—C6	119.20 (13)	C5—C14—C15	109.86 (12)
C12A—C6A—C6	119.34 (14)	C16—C14—C15	110.54 (12)

Table S10. The experimental aromatic ^{1}H chemical shifts of compounds **1**, **2**, **3**, **5** and **6**.

	Solvent	Aromatic
**1**	DMSO-d_6	7.25, 7.31, 7.41, 7.50, 7.64, 7.69, 7.73, 7.95, 8.11
**2a**	$CDCl_3$	7.17, 7.26, 7.34, 7.60

**3**	DMSO-d_6	7.54, 8.41, 8.88
**5**	$CDCl_3$	7.58, 8.21, 9.57
**6a**	$CDCl_3$	7.23, 7.51, 7.57, 8.00, 8.08, 9.48
**6b**	$CDCl_3$	7.23, 7.52, 7.57, 7.98, 8.08, 9.48
**6c**	$CDCl_3$	7.24, 7.62, 8.11, 8.13, 8.18, 9.06, 9.63
**6d**	$CDCl_3$	7.25, 7.65, 7.93, 8.03, 8.12, 8.89, 9.10, 9.28
**6e**	$CDCl_3$	7.07, 7.52, 7.93

For clarity the coupling constants are omitted.

Table S11. The experimental aromatic $^{13}C\{^{1}H\}$ chemical shifts of compounds **1**, **2**, **3**, **5** and **6**.

	Solvent	Aromatic
**1**	DMSO-d_6	124.8, 125.1, 127.2, 127.5, 127.7, 127.9, 128.0, 128.1, 128.4, 130.2, 130.3, 130.9, 134.8, 135.9, 138.6, 139.7, 145.3, 149.8, 169.5
**2a**	$CDCl_3$	123.6, 126.0, 126.8, 127.7, 127.6, 130.0, 139.5
**3**	DMSO-d_6	116.8, 120.8, 121.2, 131.2, 132.7, 136.8
**5**	$CDCl_3$	122.4, 124.2, 125.5, 133.2, 140.6, 141.3, 148.2, 151.7, 160.1
**6a**	$CDCl_3$	104.3, 121.5, 122.0, 124.1, 125.6, 129.8, 133.3, 138,2, 140.1, 141.7, 144.1, 148.7, 149.4, 151.0, 159.9
**6b**	$CDCl_3$	104.3, 121.5, 122.1, 124.0, 129.4, 129.9, 133.2, 138.2, 139.3, 140.0, 142.0, 144.1, 149.8, 151.1, 160.2
**6c**	$CDCl_3$	103.7, 121.9, 122.0, 126.7, 130.0, 130.6, 131.4, 133.3, 138.4, 139.6, 141.9, 144.4, 149.9, 151.29, 151.34
**6d**	$CDCl_3$	103.9, 118.2, 122.8, 126.9, 128.4, 129.4, 130.0, 136.4, 139.3, 141.16, 141.25, 144.7, 146.1, 150.5, 151.5
**6e**	$CDCl_3$	104.3, 122.5, 129.1, 139.6, 143.2, 149.8

For clarity the coupling constants are omitted.

Table S12. The optimized Cartesian coordinates of the **1** in the ground state calculated at the DFT/B3LYP/6-31G(d,p) level of theory

Center Number	Atomic Number	X	Y	Z
1	16	3.381161	0.842508	2.373874
2	8	5.138311	2.510752	-2.191728
3	7	2.266011	0.083748	-1.400038
4	7	3.853086	1.566673	-0.520683
5	7	1.676097	-0.834409	1.204451
6	6	0.157442	-2.460855	0.286322
7	1	-0.044953	-2.802598	1.295921
8	6	-0.459787	-3.019016	-0.813214
9	1	-1.171867	-3.828025	-0.681725
10	6	-0.176868	-2.545779	-2.117462
11	1	-0.675310	-2.998478	-2.969223
12	6	0.723994	-1.520345	-2.314220
13	1	0.960391	-1.143681	-3.303737
14	6	1.371923	-0.930606	-1.203204
15	6	2.856229	0.578589	-0.341996
16	6	3.820910	2.709418	0.348724
17	6	3.973574	4.021330	-0.117586
18	1	4.114728	4.190458	-1.174312
19	6	3.975954	5.085612	0.783703
20	1	4.112314	6.094764	0.407705
21	6	3.789191	4.864574	2.147246
22	1	3.781197	5.695811	2.845258

23	6	3.586854	3.567220	2.612531
24	1	3.405916	3.380616	3.666456
25	6	3.612616	2.490780	1.722273
26	6	2.531403	0.128530	0.984909
27	6	1.081453	-1.403578	0.112370
28	6	4.712258	1.505414	-1.649890
29	6	5.166550	0.147572	-2.091375
30	6	5.460074	-0.027599	-3.450023
31	1	5.283311	0.795959	-4.133286
32	6	5.971300	-1.239718	-3.905737
33	1	6.183533	-1.373683	-4.962258
34	6	6.218598	-2.277966	-3.004322
35	1	6.623933	-3.221113	-3.359025
36	6	5.954188	-2.098790	-1.645277
37	1	6.163774	-2.896479	-0.939040
38	6	5.426066	-0.892348	-1.188667
39	1	5.239709	-0.750838	-0.128591

Table S13. The optimized Cartesian coordinates of the **2a** in the ground state calculated at the DFT/B3LYP/6-31G(d,p) level of theory

Center Number	Atomic Number	Coordinates (Angstroms) X	Y	Z
1	15	1.635666	2.345068	7.105656
2	16	2.123670	5.985995	4.502069
3	8	2.467413	1.458587	6.049936
4	8	0.099311	2.202104	6.645891
5	8	1.871223	1.968264	8.511804
6	6	4.848026	5.648356	4.919068
7	1	4.981514	6.411466	4.158633
8	6	5.951446	5.038692	5.515541
9	1	6.953914	5.321031	5.209454
10	6	5.762611	4.085580	6.517496
11	1	6.618396	3.623605	6.999647
12	6	4.476304	3.735286	6.926148
13	1	4.326160	3.019681	7.727683
14	6	3.364808	4.307337	6.297931
15	6	0.260226	5.059037	7.987048
16	1	0.328898	4.274689	8.733486
17	6	-0.655258	6.100689	8.132455
18	1	-1.313409	6.114045	8.995541
19	6	-0.710286	7.130418	7.191910
20	1	-1.413320	7.948300	7.315027
21	6	0.156773	7.121378	6.099605
22	1	0.143581	7.930564	5.376100
23	6	1.048884	6.060586	5.927885
24	6	3.556834	5.273124	5.296764
25	7	2.024840	3.949176	6.650776
26	6	1.099051	5.018739	6.868091
27	6	2.107062	1.528938	4.646322
28	1	2.377039	2.518823	4.260663
29	1	2.730016	0.778963	4.152931
30	6	0.612265	1.242698	4.414261
31	6	-0.210881	2.256486	5.229661
32	1	-1.279215	2.037264	5.162008
33	1	-0.037999	3.276788	4.868293
34	6	0.304236	1.457297	2.922619
35	1	0.882059	0.763056	2.303627
36	1	-0.755894	1.277356	2.715865
37	1	0.543469	2.476770	2.601320
38	6	0.266187	-0.196605	4.834300
39	1	0.478276	-0.367970	5.891645
40	1	-0.795705	-0.402759	4.663225
41	1	0.846996	-0.918301	4.250181

Table S14. The optimized Cartesian coordinates of the **3** in the ground statecalculated at the DFT/B3LYP/6-31G(d,p) level of theory

Center Number	Atomic Number	Coordinates (Angstroms) X	Y	Z
1	6	-3.560394	1.533115	-0.231283
2	6	-3.636074	0.142933	-0.332716
3	6	-2.354168	2.123289	0.116032
4	6	-1.223640	1.331093	0.382099
5	6	-1.342989	-0.069001	0.313855
6	6	-2.537065	-0.668306	-0.062590
7	1	-2.613747	-1.747266	-0.140439
8	1	-4.438657	2.130099	-0.443034
9	1	-2.274381	3.206628	0.159182
10	7	-4.906851	-0.477814	-0.719700
11	8	-4.941966	-1.704898	-0.796566
12	8	-5.859629	0.270124	-0.943517
13	7	-0.000007	1.930166	0.676179
14	6	1.223630	1.331102	0.382099
15	6	1.342989	-0.068992	0.313855
16	16	0.000004	-1.153472	0.891748
17	6	2.354152	2.123306	0.116032
18	6	3.560382	1.533140	-0.231283
19	6	3.636071	0.142959	-0.332716
20	6	2.537068	-0.668288	-0.062590
21	1	2.613759	-1.747248	-0.140439
22	1	4.438641	2.130130	-0.443035
23	1	2.274358	3.206645	0.159182
24	1	-0.000011	2.942240	0.673562
25	7	4.906852	-0.477778	-0.719700
26	8	5.859625	0.270169	-0.943508
27	8	4.941977	-1.704863	-0.796553
28	8	0.000008	-2.425470	0.086265

Table S15. The optimized Cartesian coordinates of the **5** in the ground state calculated at the DFT/B3LYP/6-31G(d,p) level of theory

Center Number	Atomic Number	Coordinates (Angstroms) X	Y	Z
1	7	-0.341152	1.321275	0.367366
2	7	3.435543	1.322745	3.388064
3	6	0.712325	0.698378	0.906868
4	6	1.062485	-0.639538	0.537923
5	6	2.201550	-1.269882	1.140830
6	1	2.406262	-2.284774	0.818782
7	6	2.987590	-0.643377	2.065583
8	6	2.648695	0.720991	2.461546
9	6	1.528713	1.379295	1.891640
10	6	1.248355	2.692628	2.315338
11	1	0.394152	3.205777	1.887185
12	6	2.060572	3.287117	3.256582
13	1	1.867387	4.298643	3.601109
14	6	3.158106	2.562673	3.777570
15	6	4.065446	3.181974	4.811019
16	1	4.849652	2.475396	5.085669
17	1	3.505959	3.457704	5.712355
18	1	4.531298	4.097699	4.429085
19	6	4.213968	-1.299221	2.681074
20	1	4.256500	-0.957609	3.720671
21	6	4.165181	-2.833061	2.677039
22	1	3.247734	-3.211388	3.138874
23	1	5.015852	-3.232257	3.238536
24	1	4.228121	-3.243817	1.663208
25	6	5.499022	-0.791406	1.992225
26	1	5.511326	-1.073833	0.933534
27	1	6.383597	-1.227421	2.469321
28	1	5.575549	0.295996	2.062337

29	7	0.341152	-1.321275	-0.367366
30	7	-3.435543	-1.322745	-3.388064
31	6	-0.712325	-0.698378	-0.906868
32	6	-1.062485	0.639538	-0.537923
33	6	-2.201550	1.269882	-1.140830
34	1	-2.406262	2.284774	-0.818782
35	6	-2.987590	0.643377	-2.065583
36	6	-2.648695	-0.720991	-2.461546
37	6	-1.528713	-1.379295	-1.891640
38	6	-1.248355	-2.692628	-2.315338
39	1	-0.394152	-3.205777	-1.887185
40	6	-2.060572	-3.287117	-3.256582
41	1	-1.867387	-4.298643	-3.601109
42	6	-3.158106	-2.562673	-3.777570
43	6	-4.065446	-3.181974	-4.811019
44	1	-4.849652	-2.475396	-5.085669
45	1	-3.505959	-3.457704	-5.712355
46	1	-4.531298	-4.097699	-4.429085
47	6	-4.213968	1.299221	-2.681074
48	1	-4.256500	0.957609	-3.720671
49	6	-4.165181	2.833061	-2.677039
50	1	-3.247734	3.211388	-3.138874
51	1	-5.015852	3.232257	-3.238536
52	1	-4.228121	3.243817	-1.663208
53	6	-5.499022	0.791406	-1.992225
54	1	-5.511326	1.073833	-0.933534
55	1	-6.383597	1.227421	-2.469321
56	1	-5.575549	-0.295996	-2.062337

Table S16. The optimized Cartesian coordinates of the **6a** in the ground state calculated at the DFT/B3LYP/6-31G(d,p) level of theory

Center Number	Atomic Number	Coordinates (Angstroms) X	Y	Z
1	7	3.916985	1.003027	-0.092781
2	7	-0.767546	-1.947836	-0.083973
3	7	-0.913156	0.888554	-0.032574
4	7	-5.724534	0.620479	-0.009555
5	6	1.504699	2.409490	-0.049158
6	1	0.555860	2.934703	-0.033380
7	6	2.710531	3.078624	-0.057598
8	1	2.746935	4.163990	-0.048079
9	6	3.910502	2.332724	-0.080447
10	6	2.740948	0.327887	-0.083428
11	6	2.795642	-1.132671	-0.103277
12	6	1.621861	-1.828022	-0.107415
13	1	1.607096	-2.912021	-0.130552
14	6	0.335218	-1.188698	-0.083042
15	6	-1.947646	-1.305436	-0.059661
16	6	-3.162026	-2.049233	-0.053747
17	1	-3.092088	-3.132109	-0.067282
18	6	-4.374912	-1.418406	-0.028466
19	1	-5.275178	-2.019925	-0.021243
20	6	-4.479330	0.019630	-0.011328
21	6	-3.301024	0.763846	-0.005098
22	1	-3.300904	1.845277	0.022583
23	6	-2.035554	0.131783	-0.034049
24	6	0.253120	0.245991	-0.057330
25	6	1.493387	1.002762	-0.062035
26	6	5.248442	3.029641	-0.093407
27	1	6.050250	2.290173	-0.105139
28	1	5.369459	3.669142	0.788588
29	1	5.349301	3.674296	-0.974231
30	6	4.156384	-1.812925	-0.096332
31	1	4.814257	-1.193034	-0.714998
32	6	4.754015	-1.813533	1.327007
33	1	4.837127	-0.795904	1.715240
34	1	5.755018	-2.259146	1.319955
35	1	4.128195	-2.397278	2.011444
36	6	4.141802	-3.232639	-0.678841
37	1	3.580101	-3.930202	-0.047437

38	1	5.164997	-3.615602	-0.749669
39	1	3.701461	-3.255268	-1.680681
40	6	-6.931498	-0.170531	0.174261
41	1	-7.052236	-0.912620	-0.623369
42	1	-7.796953	0.491706	0.135506
43	1	-6.952331	-0.698703	1.138651
44	6	-5.810111	2.066070	0.109268
45	1	-5.396257	2.434982	1.059612
46	1	-6.855072	2.371717	0.047964
47	1	-5.267967	2.558128	-0.706561
